# Allantoin, a stress-related purine metabolite, can activate jasmonate signaling in a MYC2-regulated and abscisic acid-dependent manner

**DOI:** 10.1093/jxb/erw071

**Published:** 2016-03-01

**Authors:** Hiroshi Takagi, Yasuhiro Ishiga, Shunsuke Watanabe, Tomokazu Konishi, Mayumi Egusa, Nobuhiro Akiyoshi, Takakazu Matsuura, Izumi C. Mori, Takashi Hirayama, Hironori Kaminaka, Hiroshi Shimada, Atsushi Sakamoto

**Affiliations:** ^1^Graduate School of Science, Hiroshima University, Higashi-Hiroshima 739-8526, Japan; ^2^Faculty of Life and Environmental Sciences, University of Tsukuba, Tsukuba 305-8572, Japan; ^3^Faculty of Bioresource Sciences, Akita Prefectural University, Akita 010-0195, Japan; ^4^Faculty of Agriculture, Tottori University, Tottori 680-8553, Japan; ^5^Institute of Plant Science and Resources, Okayama University, Kurashiki 710-0046, Japan

**Keywords:** ABA, Arabidopsis, JA, purine metabolism, small metabolite, stress hormone, stress response, ureide.

## Abstract

Allantoin is a metabolic intermediate of purine catabolism that often accumulates in stressed plants. Recently, we used Arabidopsis knockout mutants (*aln*) of *ALLANTOINASE* to show that this purine metabolite activates abscisic acid (ABA) production, thereby stimulating stress-related gene expression and enhancing seedling tolerance to abiotic stress. A detailed re-examination of the microarray data of an *aln* mutant (*aln-1*) confirmed the increased expression of ABA-related genes and also revealed altered expression of genes involved in jasmonic acid (JA) responses, probably under the control of MYC2, a master switch in the JA signaling pathway. Consistent with the transcriptome profiles, the *aln-1* mutant displayed increased JA levels and enhanced responses to mechanical wounding and exogenous JA. Moreover, *aln* mutants demonstrated modestly increased susceptibility to *Pseudomonas syringae* and *Pectobacterium carotovorum*, probably reflecting the antagonistic action of MYC2 on the defense against these bacterial phytopathogens. Exogenously administered allantoin elicited the expression of JA-responsive genes, including *MYC2*, in wild-type plants, supporting the idea that allantoin might be responsible for the observed JA-related phenotypes of *aln* mutants. However, mutants deficient in bioactive JA (*jar1-1*), insensitive to JA (*myc2-3*), or deficient in ABA (*aba2-1* and *bglu18*) suppressed the effect of exogenous allantoin. The suppression was further confirmed in *aln-1 jar1-1* and *aln-1 bglu18* double mutants. These results indicate that allantoin can activate the MYC2-regulated JA signaling pathway through ABA production. Overall, this study suggests a possible connection of purine catabolism with stress hormone homeostasis and signaling, and highlights the potential importance of allantoin in these interactions.

## Highlight

Allantoin, a stress-related purine metabolite, can activate JA responses via ABA in Arabidopsis, suggesting its possible involvement in the homeostasis of these phytohormones and their interplay in stress signaling.

## Introduction

Plants have evolved a complex diversity of metabolic pathways to maintain and improve their growth and survival in natural environments, including stressful conditions. Plants confronted with stressful conditions typically accumulate an amazing array of small organic compounds, including metabolic products and intermediates. Numerous *in vitro* and *in vivo* studies have shown that various small metabolites serve specialized functions in cellular protection, such as scavenging reactive oxygen species (ROS), osmoregulation, stabilization of protein structure, and maintenance of membrane integrity (Chen and Murata, 2002). Certain small metabolites also play signaling and regulatory roles in mediating plant responses and adaptation to stress (see, for example, Szabados and Savouré, 2010; Wind *et al.*, 2010; Hussain *et al.*, 2011; Lunn *et al.*, 2014). However, the physiological function and significance of the accumulation of many stress-associated small compounds remain elusive.

Allantoin, a nitrogen-rich heterocyclic compound, occurs ubiquitously in plants as an intermediary metabolite of purine catabolism. Allantoin and its acyclic metabolite allantoate are collectively called ureides and, in tropical legumes, they serve as the vehicle for storage and xylem transport of symbiotically fixed nitrogen (Smith and Atkins, 2002). In non-legume plants, purine catabolism is believed to function as a fundamental route for nitrogen recycling and remobilization, since the sequential degradation of the purine ring, which initiates from oxidation of xanthine, proceeds through ureide hydrolysis and releases four molar equivalents of ammonia (Supplementary Fig. S1 at *JXB* online; Zrenner *et al.*, 2006; Werner and Witte, 2011). However, conventional metabolite analyses and recent metabolomics studies revealed allantoin as a major purine metabolite in Arabidopsis (*Arabidopsis thaliana*), rice (*Oryza sativa* L.), and other species under various stress conditions such as drought (Oliver *et al.*, 2011; Silvente *et al.*, 2011; Yobi *et al.*, 2013), high salinity (Kanani *et al.*, 2010; Wu *et al.*, 2012; Wang *et al.*, 2016), cold (Kaplan *et al.*, 2004), nutrient constraint (Nikiforova *et al.*, 2005; Rose *et al.*, 2012; Coneva *et al.*, 2014), extended darkness (Brychkova *et al.*, 2008), and pathogen invasion (Montalbini, 1991).

The possible involvement of allantoin in stress protection was initially suggested from studies on *XANTHINE DEHYDROGENASE* (*XDH*) knockout/knockdown mutants (*xdh1* and RNAi lines) of Arabidopsis. In these mutants, defects in xanthine oxidation resulted in significantly impaired stress tolerance, as well as stress-induced symptoms such as early senescence (Nakagawa *et al.*, 2007; Brychkova *et al.*, 2008; Watanabe *et al.*, 2010, 2014a). The stress-susceptible *xdh* phenotype might be at least in part attributable to purine metabolite deficiency, because supplementation with allantoin or its precursor uric acid, a product of XDH, reversed the mutant phenotype. Recent studies on knockout mutants (*aln*) of the Arabidopsis *ALLANTOINASE* gene (*ALN*) provided more direct evidence; the *aln* mutants showed constitutive accumulation of allantoin and enhanced seedling survival and growth under drought and osmotic stress (Watanabe *et al.*, 2014b). Allantoin may function in stress protection by scavenging ROS; indeed, leaf disc assays showed that exogenous allantoin alleviated ROS accumulation and cell death (Brychkova *et al.*, 2008). Allantoin may also activate stress responses, as its accumulation in *aln* mutants or supplementation of wild-type (WT) seedlings increased basal levels of the stress phytohormone abscisic acid (ABA), with concomitant activation of stress-related gene expression (Watanabe *et al.*, 2014b).

ABA has pivotal roles in plant growth and adaptive responses to various environmental stresses, mostly through antagonistic, synergistic, and additive interactions with other phytohormones such as jasmonic acid (JA), salicylic acid (SA), and ethylene. ABA, in particular, has long been known to regulate JA responses positively. JA and its metabolites, such as the bioactive metabolite jasmonoyl-l-isoleucine (JA-Ile), are collectively known as jasmonates; the jasmonates regulate diverse aspects of plant development and defense responses to insect herbivores and microbial pathogens (Wasternack and Hause, 2013). The complex interplay of the ABA and JA pathways controls defense gene expression in response to biotic stress. Early studies in potato and tomato plants reported that ABA elicits wounding responses and wound-inducible JA production (Pēna-Cortés *et al.*, 1989, 1995). Recent studies in Arabidopsis demonstrated that ABA can increase JA levels in disease conditions, and activate JA signaling for systemic induced resistance against herbivores (Adie *et al.*, 2007; Fan *et al.*, 2009; Vos *et al.*, 2013).

In Arabidopsis, the JA signaling pathway consists of two antagonistically acting branches, the ABA-co-regulated MYC branch and the ethylene-co-regulated ETHYLENE RESPONSE FACTOR (ERF) branch. The MYC branch responds to herbivore feeding and mechanical wounding, and is controlled by the basic helix–loop–helix leucine zipper transcription factors MYC2/3/4 (Anderson *et al.*, 2004; Fernández-Calvo *et al.*, 2011; Niu *et al.*, 2011). The ERF branch responds to infection with necrotrophic pathogens and is controlled by the APETALA2 (AP2)/ERF domain transcription factors ERF1 and OCTADECANOID-RESPONSIVE ARABIDOPSIS AP2/ERF 59 (ORA59) (Penninckx *et al.*, 1998; Lorenzo *et al.*, 2004; Pré *et al.*, 2008). ABA acts synergistically on the MYC branch and antagonistically on the ERF branch pathways (Anderson *et al.*, 2004).

The effects of ABA on these branches are mediated, at least in part, through transcriptional regulation by the ABA/JA-responsive MYC2 transcription factor [also known as JASMONATE INSENSITIVE1 (JAI1/JIN1)] (Anderson *et al.*, 2004; Kazan and Manners, 2013). Originally identified as a positive regulator of ABA-dependent drought responses (Abe *et al.*, 1997), MYC2 orchestrates the expression of early JA-responsive genes that play key roles in JA signaling and responses. While activating herbivore and wounding responses, MYC2 acts as a negative regulator of resistance to necrotrophic pathogens by repressing *ERF1* and *ORA59* (Lorenzo *et al.*, 2004; Dombrecht *et al.*, 2007). MYC2 also suppresses SA-dependent defenses against biotrophic pathogens and innate immune responses (Laurie-Berry *et al.*, 2006; Millet *et al.*, 2010). In addition to its antagonistic co-ordination of biotic stress responses to herbivores and pathogens, MYC2 also participates in regulation of a broad spectrum of JA-related responses, including the biosynthesis of JA and anthocyanin, JA-induced root growth inhibition, and oxidative stress tolerance (Lorenzo *et al.*, 2004; Dombrecht *et al.*, 2007; Kazan and Manners, 2013; Wasternack and Hause, 2013). Thus, MYC2 is considered a master regulator of most aspects of the JA signaling pathway as well as a hub that integrates ABA and JA signaling.

Given the critical role of JA and its intimate regulatory interaction with ABA in plant stress responses, this study investigated whether allantoin affects JA signaling and responsiveness in Arabidopsis. Detailed phenotypic and transcriptomic analyses of allantoin-accumulating *aln* mutants revealed activation of JA responses at the levels of gene expression, metabolism, physiology, and pathophysiology, in a MYC2-regulated manner. These JA-related mutant phenotypes involve allantoin because exogenous application of allantoin in WT Arabidopsis plants induced the expression of *MYC2* and MYC2-regulated JA-responsive genes. However, disrupting JA signaling or blocking ABA production abrogated the effect of both the *aln* mutation and exogenous allantoin. The results presented here thus provide evidence that allantoin can activate JA responses via ABA, and suggest that purine catabolism may have the potential to affect the homeostasis of these phytohormones and their interplay in stress signaling.

## Materials and methods

### 

#### Plant materials and growth conditions

*Arabidopsis thaliana* (L.) Heynh., accession Columbia-0, was used for all experiments. Seeds of the following mutants and T-DNA insertion lines were obtained from the Arabidopsis Biological Resource Center (Ohio State University, Columbus, OH, USA): *aah* (SALK_112631; Todd and Polacco, 2006), *aba2-1* (CS156; Léon-Kloosterziel *et al.*, 1996), *aln-1* (SALK_000325; Yang and Han, 2004; Watanabe *et al.*, 2014b; also known as *aln*), *aln-2* (SALK_146783; Watanabe *et al.*, 2014b), *bglu18* (SALK_075731C; Ogasawara *et al.*, 2009), *jar1-1* (CS8072; Staswick *et al.*, 1992), *myc2-3* (SALK_061267; identical to *jin1-8* reported in Lorenzo *et al.*, 2004), and *xdh1* (SALK_148366; Watanabe *et al.*, 2014b). The double mutants *aln-1 bglu18* and *aln-1 jar1-1* were constructed by manual cross-pollination. A homozygous complemented line of *aln-1* (*aln-1 35S:ALN*) was described in our previous study (Watanabe *et al.*, 2014b). Under standard conditions, surface-sterilized seeds were sown on solid medium containing half-strength Murashige and Skoog (1/2MS) salts, 1.0% (w/v) sucrose, and 0.3% (w/v) gellan gum. After 2 d at 4 °C to break seed dormancy, plates were placed in a growth chamber maintained at 23 °C under long-day conditions (16h light/8h dark) with 70 µmol photons m^−2^ s^−1^.

#### Gene expression analyses

For transcriptome analysis, the previously deposited raw data from the microarray data sets (NCBI Gene Expression Omnibus GSE44922) were parametrically normalized by the three-parameter lognormal distribution method (Konishi, 2004), using the SuperNORM data service (Skylight Biotech Inc., Akita, Japan). The renormalized data have been deposited under accession number GSE73841. The significance of differentially expressed genes showing ≥3-fold changes was statistically tested by a two-way ANOVA with a significance threshold set at 0.001 (Konishi, 2011). Functional enrichment analyses of the Biological Process Gene Ontology (GO) terms were carried out using the BioMaps tool of VirtualPlant version 1.3 (Katari *et al.*, 2010; http://virtualplant.bio.nyu.edu/cgi-bin/vpweb/, accessed 22 February 2016) in the default-setting mode (Fisher’s exact test with false discovery rate correction, *P*<0.01), with the Arabidopsis genome annotation (TAIR release 10; https://www.arabidopsis.org/, accessed 22 February 2016) as the background.

For quantitative transcript analysis, samples of aerial parts were collected from 2-week-old seedlings that were grown aseptically on solid 1/2MS medium with or without added allantoin (Wako Pure Chemical Industries, Ltd, Osaka, Japan) or allantoic acid (Carbosynth Limited, Berkshire, UK). RNA extraction and real-time reverse transcription quantitative PCR (RT-qPCR) were performed as described previously (Watanabe *et al.*, 2014b). Relative gene expression levels were calculated by the 2^−∆∆*C*t^ method (Livak and Schmittgen, 2001) after normalization to *ACTIN2* expression levels. Primer sequences for target genes are listed in Supplementary Table S1.

#### Quantification of JA and JA-Ile

JA and JA-Ile were quantified following the method of Preston *et al.* (2009) with minor modifications, by using the stable isotope-labeled compounds [^2^H_2_]JA and [^13^C_6_]JA-Ile as internal standards for quantitative LC-electrospray ionization-tandem MS (LC-ESI-MS/MS), as detailed in Supplementary Methods S1; Supplementary Table S2.

#### Metabolite analyses

Anthocyanin was extracted from the aerial parts of 8-day-old seedlings with methanol acidified with 1% (v/v) hydrochloric acid, and the anthocyanin level was calculated based on the absorbance at 530nm and 657nm according to Teng *et al.* (2005). Allantoin was determined in 2-week-old seedlings as described previously (Watanabe *et al.*, 2014b).

#### Root growth inhibition assay

Seeds were surface sterilized and sown onto square plates of solid 1/2MS medium containing methyl jasmonate (MeJA; Sigma-Aldrich, St Louis, MO, USA). Square plates were set vertically in a growth chamber and, 8 d after germination, the length of each primary root was measured using ImageJ version 1.45 (http://rsbweb.nih.gov/ij/, accessed 22 February 2016).

#### Wounding treatment

All rosette leaves of 2-week-old aseptically grown seedlings were wounded mechanically once by crushing with tweezers. Aerial parts of the plants were collected at the indicated time after wounding and then quick-frozen in liquid nitrogen for RNA extraction.

#### Pathogen inoculation

Surface-sterilized seeds were germinated and grown on 0.3% (w/v) Phytagel-solidified 1/2MS medium at 24 °C under a photoperiod of 12h light/12h dark with a light intensity of 150–200 µmol photons m^−2^ s^−1^. Two weeks after germination, seedlings were used for evaluation of resistance to *Pseudomonas syringae* pv. *tomato* (*Pst*) strain DC3000 according to Ishiga *et al.* (2011) and to *Pectobacterium carotovorum* subsp. *carotovorum* (*Pcc*) EC1 as described in Higashi *et al.* (2008). For *Pst* DC3000, seedlings were inoculated by flooding with a bacterial suspension [5×10^6^ colony-forming units (CFU) ml^−1^]. At 48h and 96h post-inoculation (hpi), aerial parts were collected. After being surface sterilized with 5% (v/v) H_2_O_2_, samples were homogenized in sterile water to recover internal bacteria and, upon appropriate dilution, bacterial populations were determined by colony formation on MG agar medium containing rifampicin. For infection with *Pcc* EC1, intact leaves were drop-inoculated with 5 μl of a bacterial suspension in 0.9% (w/v) NaCl; this suspension was pre-adjusted to a 600nm optical density of 0.01. The levels of disease symptoms were categorized as: level 0, no symptoms; level 1, symptoms restricted within the inoculated region; level 2, symptoms extended outside of the inoculated region.

#### Statistical analyses

All data are shown as means ± SE. Means of two groups were compared by Student’s *t*-test at a 5% level of significance with Microsoft Excel. Means of three or more groups were compared by Tukey’s multiple comparison test, which was performed with IBM SPSS statistic, version 21.0 (IBM, New York, NY, USA). Statistical difference of *Pcc* EC1 resistance was tested with Fisher’s exact test using RStudio (version 0.98; https://www.rstudio.com/, accessed 22 February 2016).

#### Accession numbers

Arabidopsis Genome Initiative numbers for the genes mentioned in this article are summarized in Supplementary Methods S1.

## Results

### The *aln* mutants show increased expression of ABA- and JA-responsive genes and decreased expression of SA-related genes

Previous work reported that normally grown 2-week-old *aln* mutant (*aln-1*) seedlings exhibited genome-wide alterations in the levels of transcripts associated with stress responses, with increased expression of ABA-responsive genes. Although not conspicuous in the GO analysis (Table 1 of Watanabe *et al.*, 2014*b*), the *aln* mutants also showed increased expression of JA response signatures including genes involved in JA metabolism and signaling, such as *13-LIPOXYGENASE* (*LOX*) and *JASMONATE ZIM-DOMAIN* (*JAZ*) *PROTEINS* (Supplementary Table S2 of Watanabe *et al.*, 2014b). For this reason, and to improve the interpretation of the transcriptome profiles, the first step in this study was to renormalize these microarray data according to a three-parameter lognormal distribution model (Konishi, 2004). This refinement of the data resulted in identification of 324 genes (211 increased, Supplementary Table S3; 113 reduced, Supplementary Table S4) with a significant change of at least 3.0-fold in their transcript levels (*P*<0.001). The physiological and functional aspects of these differentially expressed genes were assessed by GO enrichment analysis for biological process terms using VirtualPlant software (*P*<0.01). As shown in Supplementary Fig. S2 as a hierarchical tree graph, 21 out of 47 of the significantly enriched GO terms for genes with increased transcript levels were relevant to the ‘response to stimulus’ category, which included genes associated with ABA and abiotic stress responses (‘response to abscisic acid stimulus’ and ‘response to abiotic stimulus’ along with its eight descendant GO terms), and also genes associated with JA-related stress responses (‘response to jasmonic acid stimulus’, ‘response to wounding’, ‘defense response to fungus’, and ‘response to biotic stimulus’). Moreover, ‘jasmonic acid metabolic process’ was identified as significantly enriched in the ‘cellular process’ category. For genes with reduced transcript levels, more than half (20 out of 33) of the significantly enriched GO terms belonged to three related categories (‘response to stimulus’, ‘multi-organism process’, and ‘immune system process’), which emphasized biotic defense responses mainly involving SA, such as innate and acquired immunity (Supplementary Fig. S3). This downward shift in basal expression levels of SA-responsive genes was confirmed by RT-qPCR of the canonical SA-marker *PATHOGENESIS-RELATED PROTEIN 1* (*PR-1*) (Supplementary Fig. S4). Taken together, the results of re-examining the transcriptome profiling suggested that the *aln* mutation caused increased transcript levels of ABA- and JA-responsive genes but decreased transcript levels of SA-related genes at the seedling stage under normal growth conditions.

### The *aln* mutants show altered basal expression of *MYC2* and MYC2-regulated genes in JA metabolism, signaling, and responses

**Fig. 1.**
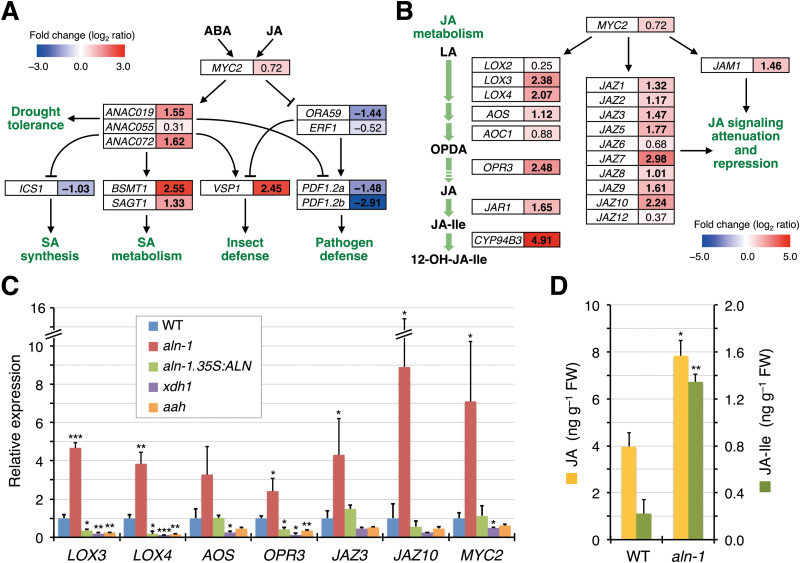
The *aln-1* mutant shows altered levels of basal expression of MYC2-regulated JA-related genes and has enhanced JA and JA-Ile levels. (A and B) Simplified schemes of MYC2-mediated signaling processes, with emphasis on JA–ABA and JA–SA crosstalk in abiotic and biotic responses (A; mostly based on Kazan and Manners, 2013) and MYC2-mediated transcriptional activation of genes for JA metabolic enzymes and those involved in JA signaling (B). Genes are indicated in open boxes, and their positive and negative actions are denoted by lines with an arrow and a bar end, respectively. For each gene, the microarray-based relative expression ratio (*aln-1* mutant versus the WT) is represented in log_2_ scale and by a boxed color on the blue-to-red gradient scale, with significant fold changes (|log_2_(*aln-1*/WT)| ≥ 1) in bold. Abbreviations not defined in the text are: LA, linolenic acid; OPDA, 12-oxo-phytodienoic acid; 12-OH-JA-Ile, 12-hydroxyjasmonoyl-l-isoleucine. (C) RT-qPCR for expression of the selected genes in the WT, three purine catabolic mutants (*aln-1*, *xdh1*, and *aah*), and the complemented mutant (*aln-1 35S:ALN*). RNA was extracted from the aerial parts of 2-week-old seedlings grown under normal aseptic conditions. Relative mRNA levels for each gene were determined using *ACTIN2* expression as reference and are presented as values relative to those of the WT. (D) Endogenous jasmonate levels of WT and *aln-1* mutants were quantified by LC-ESI-MS/MS in 2-week-old seedlings grown under normal aseptic conditions. In (C) and (D), data are means ±SE from three independent experiments, and asterisks denote significant differences between WT and mutant plants (*P<0.05; **P<0.01; ***P<0.001 by Student’s *t*-test).

Next, mapping of the relevant microarray-derived expression data onto the current model of the MYC2-modulated JA signaling pathways was conducted to obtain a more detailed picture of JA-associated gene expression in the *aln-1* mutant (Kazan and Manners, 2013), with emphasis on the crosstalk with ABA and SA (Fig. 1A). In the ABA-co-regulated MYC branch, this mapping revealed a significant increase in basal expression levels of two of the three genes encoding Arabidopsis NAM/ATAF/CUC (ANAC) transcription factors, *ANAC019*, *ANAC055*, and *ANAC072*, immediate target genes positively regulated by MYC2 (Bu *et al.*, 2008). Transcript levels of downstream MYC branch marker genes that these ANAC transcription factors regulate, positively or negatively, also showed major changes. Genes with increased transcript levels included the marker *VEGETATIVE STORAGE PROTEIN 1* (*VSP1*) along with *SABATH METHYLTRANSFERASE* (also known as *BSMT1*) and *SALICYLIC ACID GLUCOSYLTRANSFERASE 1* (*SAGT1*), both of which encode SA-inactivating enzymes. Genes with decreased transcript levels included *ISOCHORISMATE SYNTHASE 1* (*ICS1*), which encodes an SA biosynthetic enzyme. For ERF branch genes, markedly reduced basal expression was observed for the key transcription factor gene *ORA59* whose expression is negatively regulated by MYC2 (Dombrecht *et al.*, 2007), and the ORA59 downstream target and representative ERF branch marker *PLANT DEFENSIN 1.2* (*PDF1.2a* and *PDF1.2b*). Overall, the altered expression of these JA and SA markers was in accordance with the known modes of MYC2 action on its direct and downstream target genes.

MYC2 also acts as a transcriptional activator of JA-responsive genes encoding JA metabolic enzymes such as LOX, allene oxide synthase (AOS), allene oxide cyclase (AOC), 12-oxophytodienoate reductase (OPR), jasmonate-amido synthetase (also known as JASMONATE RESISTANCE1; JAR1), and cytochrome P450 monooxygenase 94B3 (CYP94B3) (Lorenzo *et al.*, 2004; Sasaki-Sekimoto *et al.*, 2013). MYC2 also activates genes encoding JA signaling components including JAZ and JASMONATE-ASSOCIATED MYC2-LIKE1 (JAM1) repressor proteins (Chini *et al.*, 2007; Nakata *et al.*, 2013; Sasaki-Sekimoto *et al.*, 2013). As shown by microarray analysis (Fig. 1B), the basal expression levels of most of these genes significantly increased in the *aln-1* mutant. This was confirmed by RT-qPCR for selected genes (*LOX3*, *LOX4*, *AOS*, *OPR3*, *JAZ3*, *JAZ10*, and *MYC2*) using a separate set of RNA samples from those used for microarray analysis (Fig. 1C). The RT-qPCR clearly demonstrated the significant activation of *MYC2* expression, although the microarray analysis only showed a modest increase in the *MYC2* transcript level (Fig. 1A, B). Genetic complementation of the *aln-1* mutant with the WT *ALN* cDNA (*aln-1 35S:ALN*) resulted in the reversion of the transcript levels of all these genes to the normal WT levels, corroborating the effect of the mutation.

The increased expression of these JA-responsive genes could be caused by general inhibition of the purine catabolic pathway, rather than by the specific blockage of allantoin degradation. To exclude this possibility, two other knockout mutations, *xdh1* and *aah* (*allantoate amidohydrolase*), were examined which impair the metabolic steps upstream and downstream of allantoin degradation, respectively (Supplementary Fig. S1). The basal expression levels of JA-responsive genes in *xdh1* and *aah* mutants were comparable with or lower than those of the WT (Fig. 1C), supporting the idea that the *aln* mutation preventing allantoin degradation was indeed responsible for the observed gene expression phenotype.

### The *aln* mutants have increased levels of endogenous JA and JA-Ile

Based on the observation that expression of genes involved in JA metabolism was significantly increased in the *aln-1* mutant (Fig. 1B, C), endogenous JA and JA-Ile levels were measured in the WT and the mutant by LC-ESI-MS/MS (Fig. 1D). The concentrations of JA and JA-Ile in WT seedlings (4.0±0.6ng g^−1^ FW and 0.22±0.12ng g^−1^ FW, respectively) were similar to those reported previously (Pan *et al.*, 2010). Compared with WT seedlings, the *aln-1* mutant showed a 2-fold increase in JA levels and a 6-fold increase in JA-Ile levels. This increase in jasmonates correlated with the observed expression profiles (Fig. 1B, C), suggesting that the *aln* mutation promotes jasmonate accumulation in normally grown Arabidopsis seedlings, probably through the transcriptional activation of JA biosynthetic genes.

### The *aln* mutants show enhanced sensitivity to exogenous jasmonate

**Fig. 2.**
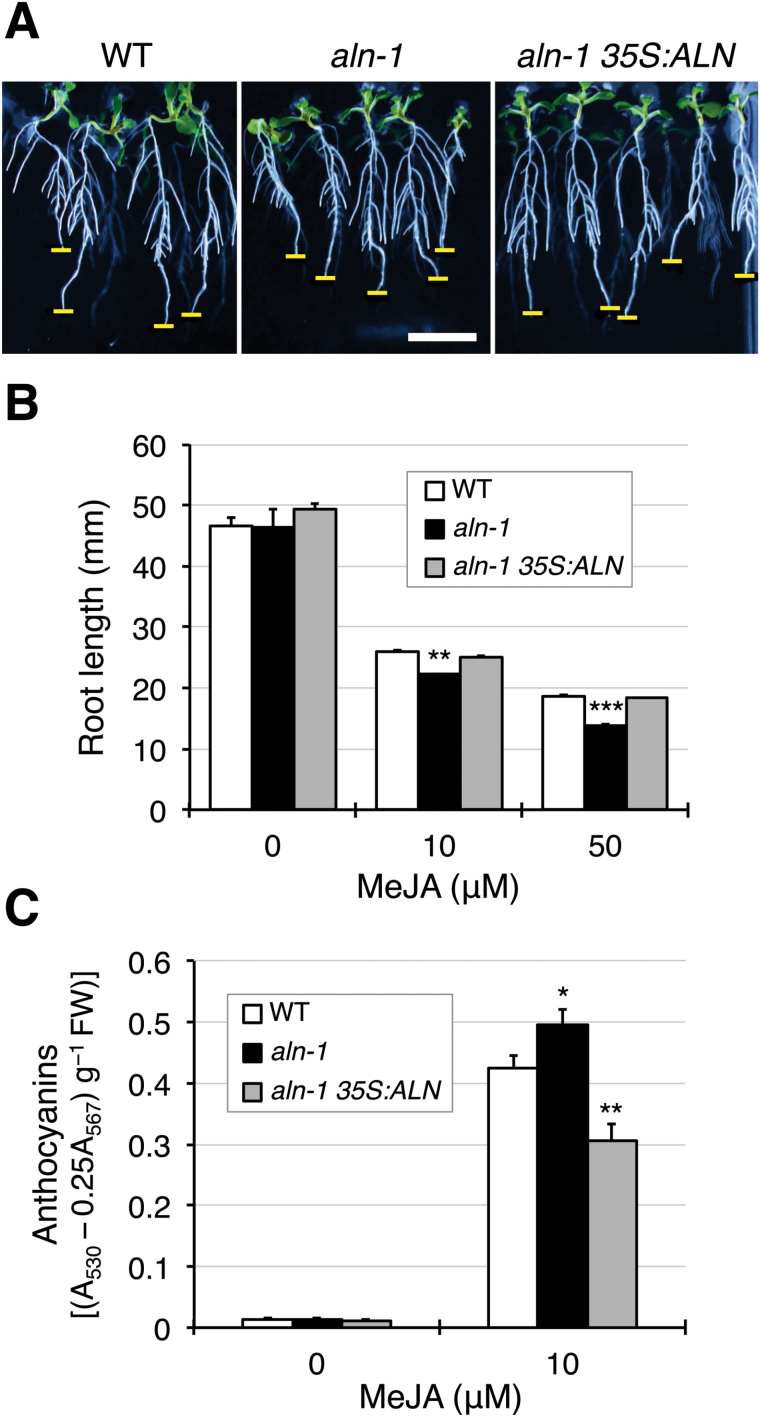
The *aln-1* mutant exhibits increased sensitivity to exogenous MeJA. Sterile seedlings of the WT, *aln-1* mutant, and the complemented mutant (*aln-1 35S:ALN*) were grown for 8 d on standard medium supplemented with MeJA and examined for root growth and anthocyanin accumulation. (A) Typical root growth in the presence of 10 µM MeJA. Horizontal bars indicate a scale of 10mm in length (thick) and the position of a primary root tip (thin). (B) Primary root length in the presence of 10 µM and 50 µM MeJA. Data are means ±SE (*n*≥16). (C) Anthocyanin levels in the presence of 10 µM MeJA. Data are means ±SE (*n*=8). Asterisks denote significant differences between WT and mutant plants (*P<0.05; **P<0.01; ***P<0.001 by Student’s *t*-test). (This figure is available in colour at *JXB* online.)

In addition to JA biosynthesis, MYC2 also positively regulates jasmonate-induced root growth inhibition and anthocyanin production (Dombrecht *et al.*, 2007). To determine whether the *aln* mutants show increased activation of these MYC2-modulated JA responses, the sensitivity of the WT, *aln-1* mutants, and the complemented line to exogenous MeJA was tested. When these genotypes were grown for 8 d on medium containing MeJA, they seemed to develop lateral roots well (Fig. 2A), probably reflecting the stimulatory effect of MeJA (Raya-González *et al.* 2012). Simultaneously, the *aln-1* seedlings showed a slight but statistically significant reduction in primary root length, compared with the WT and the complemented mutant (Fig. 2B). However, when the *aln* mutation was introduced into the JA-insensitive *jar1-1* mutant, which is defective in JA-Ile production (Staswick *et al.*, 1992), the resultant *aln-1 jar1-1* double mutant displayed no change in root growth in the presence of MeJA (Supplementary Fig. S5). Similar to the results of root growth inhibition, the *aln-1* mutant responded more strongly than the WT to exogenous MeJA in anthocyanin production, further increasing its anthocyanin accumulation in response to MeJA treatment (Fig. 2C). Conversely, the complemented line was the least responsive to MeJA treatment as it had lower levels of anthocyanin than the WT and the *aln-1* mutant. The *aah* mutant also compromised the metabolic response to MeJA (Supplementary Fig. S6). These results support the idea that the *aln* mutation enhances the MYC2-mediated physiological and metabolic responses to JA.

### The *aln* mutants show enhanced wounding-inducible expression of JA-responsive genes

**Fig. 3.**
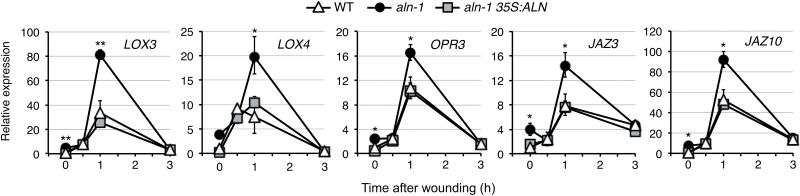
The *aln-1* mutant enhances wounding-inducible expression of genes involved in JA biosynthesis and signaling. RNA was extracted from aerial parts of 2-week-old seedlings of the WT, *aln-1* mutant, and the complemented mutant (*aln-1 35S:ALN*) at the indicated time points after wounding of rosette leaves. Relative mRNA levels were determined as described in Fig. 1C and are presented as values relative to those of the WT at zero time. Data are means ±SE from three independent experiments, and asterisks denote significant differences between WT and mutant plants (*P<0.05; **P<0.01 by Student’s *t*-test).

Next, this study investigated whether the *aln* mutation has a stimulatory effect on MYC2-mediated JA responses under stress conditions known to activate the JA signaling pathway. Wounding stress strongly enhances the transcript levels of genes involved in JA biosynthesis and signaling in a MYC2-dependent manner (Reymond *et al.*, 2000; Lorenzo *et al.*, 2004; Chung *et al.*, 2008). Thus, the expression of these JA-responsive genes (*LOX3*, *LOX4*, and *OPR3* for JA biosynthesis and *JAZ3* and *JAZ10* for JA signaling) was monitored in wounded leaves of 2-week-old seedlings (Fig. 3). Although the transcripts for these genes accumulated in response to wounding, in all cases the maximum levels were significantly higher in the mutant than in the WT, possibly reflecting the increased basal expression levels. The complemented mutant showed essentially the same transcript expression profiles as those observed in the WT. These results suggest that the *aln* mutation activates the MYC2-mediated JA response in normal growth conditions and under JA-associated stress conditions.

### The *aln* mutation affects basal resistance to *Pseudomonas syringae* DC3000

**Fig. 4.**
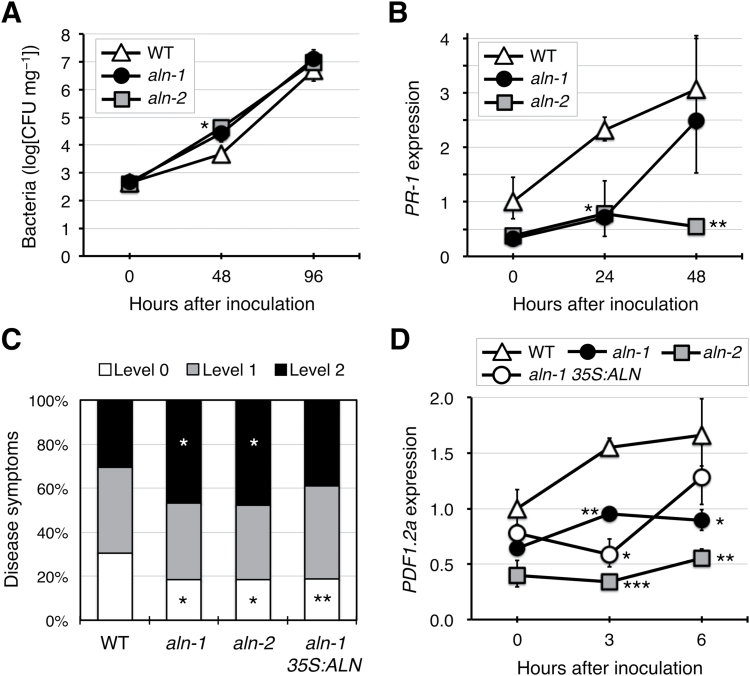
Effect of *aln* mutations on resistance to bacterial pathogens. (A) Resistance to *Pst* DC3000 was evaluated for 2-week-old seedlings of the WT and two allelic *aln* mutants (*aln-1* and *aln-2*). At 48 and 96 hpi, internal bacterial populations were determined as mg^-1^ FW of whole rosettes (*n*=4). (B) Time-course expression of the SA marker *PR-1* in leaves inoculated with *Pst* DC3000. Relative mRNA levels were determined as described in Fig. 1C and are presented as values relative to those of the WT at zero time. Data are means ±SE from three independent experiments. (C) Resistance to *Pcc* EC1 was evaluated for leaves from 2-week-old seedlings of the WT, two allelic *aln* mutants, and the complemented mutant (*aln-1 35S:ALN*). Disease symptoms were scored at 24 hpi according to three criteria: level 0, no symptoms; level 1, symptoms restricted within the inoculated region; level 2, symptoms extended over the inoculated region (*n*≥169). (D) Time-course expression of the ERF branch marker *PDF1.2a* in leaves inoculated with *Pcc* EC1 was determined as described in (B). Asterisks denote significant differences between WT and mutant plants [*P<0.05; **P<0.01; ***P<0.001 by Student’s *t*-test, except in (C), where Fisher’s exact test was used].

MYC2 negatively regulates the SA signaling pathway and suppresses SA-mediated host defenses against the bacterial pathogen *Pst* DC3000 (Nickstadt *et al.*, 2004; Laurie-Berry *et al.*, 2006). Because the gene expression data described above suggested that the *aln* mutation led to attenuation of SA signaling through activation of MYC2 (Fig. 1A; Supplementary Figs S3, S4), the resistance of two *aln* mutant alleles, *aln-1* and *aln-2*, to *Pst* DC3000 was examined by monitoring bacterial growth in inoculated seedlings (Fig. 4A). At 48 hpi, bacterial growth was evident in both the WT and the mutants, but the *aln-2* mutants had significantly more bacteria (11-fold, *P*<0.05) than the WT. Although not statistically significant, the same tendency was observed in the *aln-1* mutant (a 9-fold higher population; *P*=0.068). At 96 hpi, bacterial populations showed further increases, but no longer differed between the WT and the *aln* mutants. Simultaneous monitoring of expression of the SA marker *PR-1* during the course of infection with *Pst* DC3000 gave overall results consistent with those of the infection experiments, as induction of *PR-1* expression was compromised in the *aln-1* and *aln-2* mutants (Fig. 4B). These results suggest that the *aln* mutation reduced the resistance to *Pst* DC3000 at early stages of pathogen infection, probably due to constitutive activation of the MYC branch of JA signaling.

### The *aln* mutation suppresses basal resistance against *Pectobacterium carotovorum*

MYC2 down-regulates the ERF branch of JA signaling, which is critical for Arabidopsis resistance to phytopathogens such as *Pcc* EC1 (Norman-Setterblad *et al.*, 2000; Hiruma *et al.*, 2011). To test if the *aln* mutation altered resistance to this bacterium, *Pcc* EC1 was drop-inoculated onto intact leaves of 2-week-old seedlings and disease symptoms were evaluated at 24 hpi. As shown in Fig. 4C, the *aln-1* and *aln-2* mutants had compromised resistance to *Pcc* EC1, showing a significantly higher proportion of level 2 (most serious) symptoms and a lower proportion of level 0 (no detectable) symptoms, compared with the WT. The complemented *aln-1* mutant plants showed substantially recovered disease resistance, with a proportion of level 2 symptoms non-significantly different from the WT. The expression levels of the disease resistance gene *PDF1.2a*, a marker of the ERF branch, were also examined at early stages of infection (0, 3, and 6 hpi). As expected from the enhanced susceptibility to *Pcc* EC1, the *aln* mutants showed consistently lower levels of *PDF1.2a* expression compared with the WT (Fig. 4D). For unknown reasons, complementation of the *aln-1* mutant failed to restore *PDF1.2a* expression to the WT levels, which might explain why the resistance of the complemented mutant to *Pcc* EC1 was not fully recovered. Nevertheless, these results showed the compromised resistance of the *aln* mutants to *Pcc* EC1, providing more evidence for *aln*-mediated activation of MYC2.

### Allantoin activates expression of *MYC2* and MYC2-modulated JA-responsive genes

**Fig. 5.**
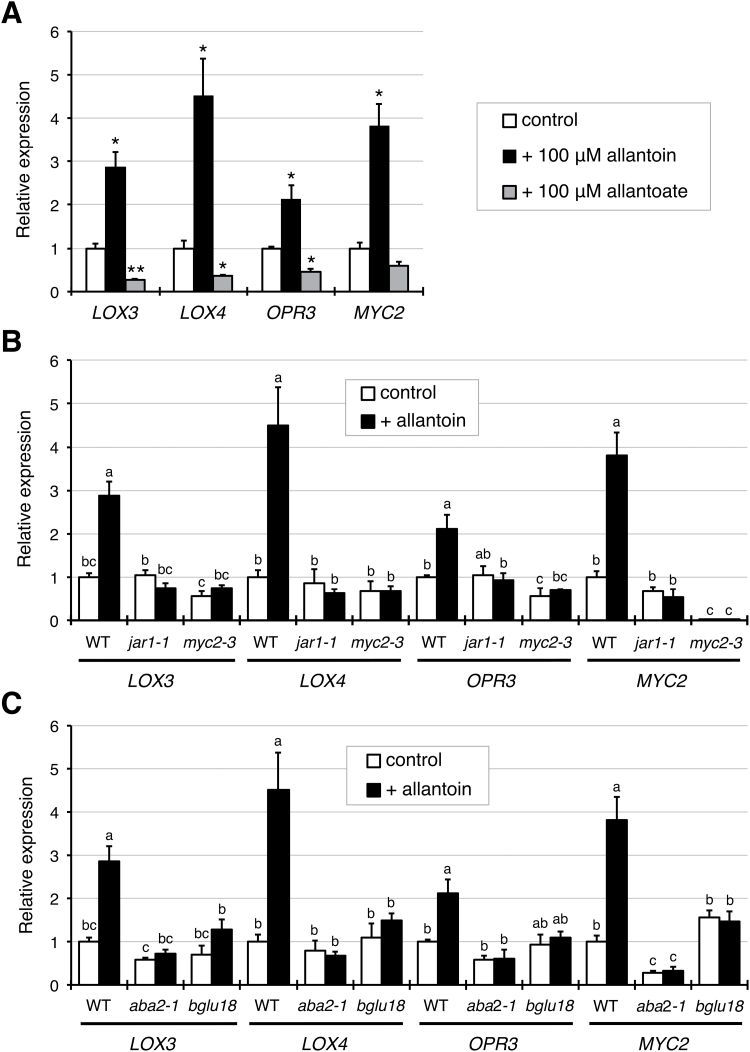
Exogenous allantoin induces JA-responsive gene expression in an ABA-dependent manner. (A) Effects of exogenous allantoin and allantoate on JA-responsive gene expression in the WT. RNA was extracted from aerial parts of sterile seedlings that had been grown for 2 weeks on standard medium containing allantoin or allantoate at 100 µM, and relative mRNA levels for each gene were determined as described in Fig. 1C. Data are means ±SE from three independent experiments, and asterisks denote significant differences between control and treated seedlings (*P<0.01; **P<0.001 by Student’s *t*-test). (B and C) Effect of exogenous allantoin on JA-responsive gene expression in bioactive JA-deficient *jar1-1* and JA-insensitive *myc2-3* mutants (B), and in ABA-deficient *aba2-1* and *bglu18* mutants (C). WT data are the same as presented in (A) and are shown for comparison. Data are means ±SE from three independent experiments, and different letters indicate significant differences (*P*<0.05 by Tukey’s test).

To address whether the observed JA-related *aln* phenotype was attributable to the accumulation of allantoin as a result of the *aln* mutation, the effects of exogenous allantoin on expression of *MYC2* and the MYC2-modulated JA-responsive genes *LOX3*, *LOX4*, and *OPR3* were examined. When seedlings of the WT were grown aseptically for 2 weeks on standard medium containing 100 µM allantoin, the four genes showed significantly increased transcript levels, compared with the non-treated control (Fig. 5A). Allantoate, which is similar in structure to allantoin as the immediate downstream metabolite, was also tested. When exogenously supplied at 100 µM, allantoate did not affect transcript levels, or caused a decrease in transcript levels (Fig. 5A), further supporting the specific efficacy of allantoin in inducing these JA-responsive genes. These results supported the idea that the JA-related *aln* mutant phenotypes result from accumulation of allantoin.

### Allantoin requires ABA to activate JA-responsive genes

To gain insight into how allantoin activates JA-dependent responses, the next step was to investigate the effects of exogenous allantoin on JA-responsive gene expression in the two mutants, JA-insensitive *jar1-1* and MYC2-deficient *myc2-3*. In marked contrast to the WT, both *jar1-1* and *myc2-3* mutants showed no responses to allantoin (Fig. 5B), demonstrating that the action of allantoin relies on an intact JA signaling pathway and requires the formation of bioactive JA-Ile. Because *MYC2* is responsive to ABA (Abe *et al.*, 1997), and because ABA levels increase in the *aln* mutants and in response to exogenous allantoin in WT plants (Watanabe *et al.*, 2014b), expression of JA-responsive genes was also examined in the ABA-deficient mutants *aba2-1* (*abscisic acid deficient 2-1*) and *bglu18* (*ß-glucosidase 18*), which impair *de novo* ABA synthesis and regeneration of ABA from inactive ABA glucosides, respectively (Léon-Kloosterziel *et al.*, 1996; Lee *et al.*, 2006; Ogasawara *et al.*, 2009). Similar to the JA-insensitive mutants, neither *aba2-1* nor *bglu18* mutants showed increases in the transcript levels of JA-responsive genes in response to exogenous allantoin (Fig. 5C).

**Fig. 6.**
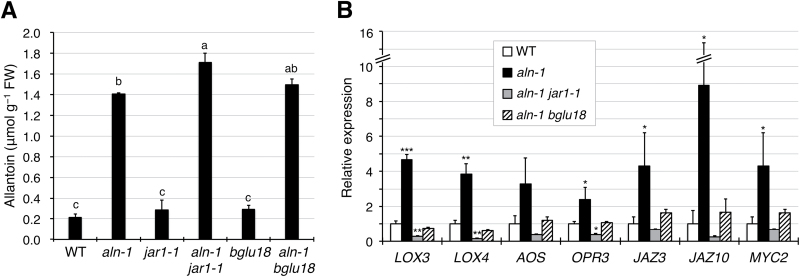
The *aln* mutation activates JA-responsive gene expression in an ABA-dependent manner. (A) Endogenous allantoin levels in aseptically grown 2-week-old seedlings of JA- and ABA-related single and double mutants. Data are means ±SE from three independent experiments, and different letters indicate significant differences (*P*<0.05 by Tukey’s test). (B) RT-qPCR was performed for RNA samples extracted from the aerial parts of 2-week-old sterile seedlings of *aln-1 jar1-1* or *aln-1 bglu18* double mutants. Relative mRNA levels for each gene were determined and are presented as values relative to WT data that are taken from Fig. 1C, to simplify comparison. Data are means ±SE from three independent experiments, and asterisks denote significant differences between WT and mutant plants (*P<0.05; **P<0.01; ***P<0.001 by Student’s *t*-test).

To obtain further evidence supporting this, the next experiments tested if these JA- and ABA-related mutations also suppress the effect of allantoin accumulation in the *aln-1* background. For this, the *aln-1* mutant was crossed with the *bglu18* mutant to establish the *aln-1 bglu18* double mutant (Supplementary Fig. S7), and the transcript levels of seven JA-responsive genes (the same set as reported in Fig. 1C) were measured in the *aln-1 jar1-1* and *aln-1 bglu18* double mutants. Although these double mutants exhibited allantoin levels comparable with the *aln-1* mutant (Fig. 6A), the expression levels of seven JA-responsive genes no longer increased significantly (Fig. 6B). Thus, ABA may be required for activation of JA responses by allantoin.

## Discussion

Evidence for the involvement of purine catabolism in stress protection in plants has only recently emerged (Brychkova *et al.*, 2008; Watanabe *et al.*, 2010), and the mechanism behind this remains unclear. Previous studies using Arabidopsis mutants defective in purine catabolism showed that the intermediary metabolite allantoin stimulated ABA production and enhanced abiotic stress tolerance (Watanabe *et al.*, 2010, 2014a, *b*). To gain more insight into the relationship between this purine metabolite and stress hormones, the present study investigated the effects of allantoin on JA signaling and responses, because the interplay between ABA and JA constitutes a fundamental regulatory mechanism in plant defense reactions (Kazan and Manners, 2013; Wasternack and Hause, 2013). The current results show that allantoin can activate JA metabolism, signaling, and responses via mechanisms involving ABA, which is supported by several lines of evidence. First, with the improved microarray data using a superior renormalization technique and a stringent cut-off threshold (Konishi, 2004, 2011), GO-based over-representation analysis of the *aln-1* mutant, which accumulates allantoin, revealed a strong effect on JA responses (Supplementary Fig. S2). This was supported by the significantly increased levels of JA and JA-Ile in the *aln-1* mutants (Fig. 1D). This analysis also reproduced the enhanced responses to ABA and abiotic stress conditions that were previously observed with the *aln-1* mutant (Watanabe *et al.*, 2014b), indicating that the *aln* mutation could cause the activation of both JA and ABA responses. Such transcriptome profiles provided a plausible explanation for the simultaneous suppression of SA responses (Fig. 1A; Supplementary Figs S3, S4), since antagonistic action of JA and ABA on SA signaling has been reported (Laurie-Berry *et al.*, 2006; Yasuda *et al.*, 2008; Millet *et al.*, 2010). Secondly, the detailed transcriptome profiling and RT-qPCR analyses emphasized the massive influence of MYC2, a master regulator of JA signaling, on global gene expression in this mutant (Fig. 1A−C). As discussed below, this finding was supported by significant alterations in metabolic, physiological, and pathophysiological responses that are mainly controlled by MYC2 (Figs 2−4). Most of these alterations were reversed upon genetic complementation of the *aln* mutation (Figs 1C, 2, 3, 4C, D), while the other mutations that disrupt different steps in purine degradation had little effect on activating JA-responsive genes (Fig. 1C). These results confirmed the *aln* mutation as the primary cause behind the observed phenotype, and ruled out non-specific effects of inhibiting purine catabolism. Thirdly, exogenously supplied allantoin, but not its immediate metabolite allantoate, activated expression of JA-responsive genes in the WT plants (Fig. 5A). The observation is consistent with the expression data for *aln-1* and *aah* mutants, which endogenously accumulate allantoin and allantoate, respectively (Fig. 1C). It thus appears that the accumulation of allantoin as a result of the *aln* mutation indeed triggers JA responses. Finally, not only JA-insensitive mutants, but also ABA-deficient mutants, showed no effect of allantoin that was either exogenously supplied or endogenously accumulated due to the *aln-1* mutation (Figs 5B, C, 6). These findings revealed the requirement for ABA in the action of allantoin to elicit JA responses, suggesting that allantoin may exert its effects through the interplay between the two phytohormones.

In Arabidopsis, the MYC and ERF branches of the JA signaling pathway play critical roles in JA-mediated defense responses, and their antagonistic interaction is co-ordinated mainly by the MYC2 transcription factor (Kazan and Manners, 2013). Surprisingly, the *aln* mutation resulted in highly contrasting effects on these two major branches, activating the MYC branch while repressing the ERF branch (Fig. 1A). This finding suggested that MYC2 activity was augmented in the *aln-1* mutant. Indeed, the *aln-1* mutant showed significantly increased *MYC2* transcript levels, which most probably accounted for transcriptional activation of MYC2 target genes (Fig. 1A−C). Consistent with these observations, *aln-1* mutants also showed enhanced sensitivity to exogenous MeJA and mechanical wounding (Figs 2, 3), which are established MYC2-mediated responses. Modestly compromised resistance of *aln* mutants to bacterial pathogens (Fig. 4) also seems to result from high levels of *MYC2* expression, perhaps due to suppression of SA signaling and the ERF branch of JA signaling (Fig. 1A). Some features of the *aln* mutant phenotype, such as enhanced sensitivity to exogenous JA, coincided with the phenotypes of transgenic Arabidopsis plants expressing *MYC2* under a strong constitutive promoter (Lorenzo *et al.*, 2004; Dombrecht *et al.*, 2007). All together, these phenotypes of *aln* mutants can be interpreted as the consequences of MYC2 activation, suggesting that allantoin may activate JA responses by enhancing expression of this master regulator.

**Fig. 7.**
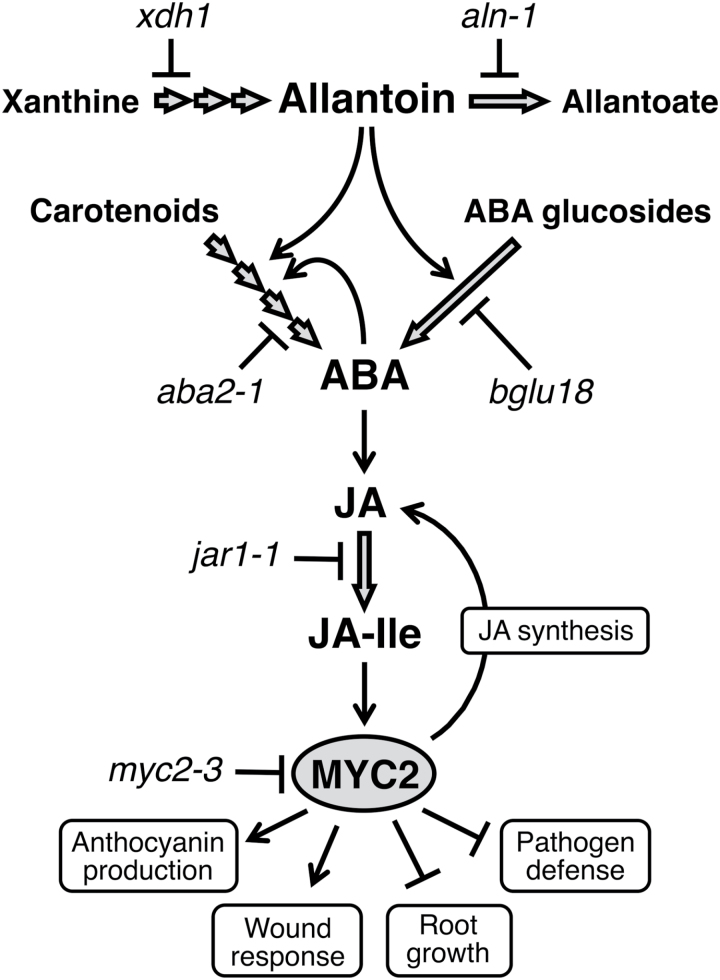
Schematic model for how allantoin activates JA responses in Arabidopsis. Accumulation of allantoin causes enhanced ABA levels through *de novo* synthesis from carotenoids and/or hydrolytic conversion from inactive ABA glucosides, thereby activating ABA-dependent JA responses mediated by MYC2. The effect of allantoin on *de novo* synthesis may be indirect due to positive feedback of ABA released from the inactive pool (Watanabe *et al.*, 2014*b*). The open arrows indicate metabolic routes, and lines with arrows and bar ends represent positive and negative causal relationships, respectively.

Arabidopsis *MYC2* is rapidly induced by ABA (Abe *et al.*, 1997), and it has been shown that ABA acts upstream of JA to regulate *MYC2* expression (Lorenzo *et al.*, 2004). Previous work showed that allantoin could increase ABA levels in Arabidopsis seedlings via two possible mechanisms: by stimulating the expression of a rate-limiting enzyme (9-*cis*-epoxycarotenoid dioxygenase 3) in *de novo* synthesis; and by post-translational activation of an enzyme (BGLU18 also known as BG1) responsible for hydrolytic conversion of inactive ABA glucosides (Watanabe *et al.*, 2014b). Therefore, allantoin might activate JA responses through ABA-dependent *MYC2* activation, although in Arabidopsis the *aln* mutation or exogenous allantoin increases ABA only at modest levels (Watanabe *et al.*, 2014b) and increased ABA levels do not always result in significantly enhanced JA responses (García-Andrade *et al.*, 2011). Here, this possibility was examined by quantifying expression of *MYC2* and MYC2-regulated genes in ABA-deficient single mutants treated with exogenous allantoin, and in ABA-deficient *aln-1* double mutants. The results revealed ABA to be an essential factor in allantoin-mediated *MYC2* activation (Figs 5C, 6B). The similar expression analysis using the *jar1-1* single and *aln-1 jar1-1* double mutants gave the same results as those for the ABA mutants (Figs 5B, 6B), indicating that the effect of ABA depends on the formation of bioactive JA (JA-Ile). These results, together with those of a previous study (Watanabe *et al.*, 2014b), support the idea that ABA might precede JA in the activation of *MYC2* by allantoin, although how ABA enhances JA remains to be elucidated (Fig. 7).

Whether the activated JA response contributes to allantoin-mediated stress tolerance, as observed in the *aln* mutants (Watanabe *et al.*, 2014*b*), remains an important question regarding the mechanism of action of allantoin. Although ABA plays a central role in drought tolerance, JA participates in the regulation of ABA-mediated physiological responses to drought such as stomatal closure (Suhita *et al.*, 2004). JA also affects expression of a significant fraction of drought-related genes that are responsive to ABA (Huang *et al.*, 2008) and positively regulates freezing tolerance (Hu *et al.*, 2013), which may share common protective mechanisms with drought tolerance. In fact, allantoin-accumulating *aln* mutants enhance ABA levels, activate stress-related genes, and increase drought and osmotic stress tolerance (Watanabe *et al.*, 2014*b*); the results described here show that the mutants also activate JA responses and JA-related gene expression. These observations may suggest a possible contribution of JA signaling to the abiotic stress tolerance mediated by allantoin, through interactions with ABA.

These results point to a previously unrecognized connection between allantoin and JA signaling, and imply its potential influence on the interplay between JA and SA (Figs 1A, 4; Supplementary Figs S3, S4). This suggests that purine catabolism may play a role in biotic stress responses. Indeed, purine catabolism has been implicated in plant–microbe interactions: in beans and wheat, changes in the enzyme activities and metabolites, including allantoin, occur as an early response to pathogens (Montalbini, 1991, 1992*b*). Also, inhibition of the purine catabolic pathway by the XDH inhibitor allopurinol resulted in altered rust infection and compromised hypersensitive responses during incompatible interactions (Montalbini, 1992a), and the application of allopurinol enhanced virus susceptibility in tobacco (Silvestri *et al.*, 2008). In addition, a recent transcriptome analysis of wheat revealed significantly increased expression of genes involved in ureide transport during fusarium infection (Lysøe *et al.*, 2011). Although the above pathophysiological phenomena may be relevant to JA and SA signaling, further research is needed to obtain evidence that substantially supports the role of purine catabolism in biotic defense. In this context, it would be interesting to examine the effect of allantoin on herbivore resistance because the *aln* mutants activated the MYC branch pathway and enhanced wounding responses (Figs 1, 3).

An intriguing observation is that *aah* mutation and exogenous allantoate treatment, both causing allantoate accumulation, tend to suppress JA responses at the transcript and metabolite levels (Figs 1C, 5A; Supplementary Fig. S6). The *aah* mutation also tends to perturb the expression of certain ABA-related genes, although the ABA level in this mutant does not seem to be significantly different from that of the WT (Watanabe *et al.*, 2014*b*). These observations may imply that allantoate also affects stress-related gene expression, potentially with the opposite effect to that of allantoin reported here and previously (Watanabe *et al.*, 2014*b*). If such a possibility were likely, then allantoate could ensure the elimination of the effect of allantoin after its degradation. However, this remains to be investigated. A more extensive analysis, including further examination of the physiological effects of ureide accumulation and in-depth characterization of *aln* and *aah* mutants, would shed light on the possible relationship between allantoin and allantoate in the mechanism of stress response and adaptation.

Recent metabolomic studies showed that allantoin levels significantly increased in Arabidopsis, rice, and other species subjected to various stress conditions (see the Introduction). Some possibilities have been proposed to explain the physiological significance of stress-induced allantoin accumulation. The conventional view argues that this phenomenon results from a metabolic counter-balance to optimize carbon to nitrogen ratios, because, under stress conditions, reduced photosynthesis causes a shortage of carbon skeletons, which restricts assimilation of purine-derived ammonia and leads to accumulation of this toxic nitrogen compound. However, recent studies using Arabidopsis mutants indicated that allantoin might participate in stress protection and tolerance mechanisms. Brychkova *et al.* (2008) reported that this ureide compound could act as an antioxidant. Our previous and current studies showed that allantoin elicited phytohormone-mediated stress responses (Watanabe *et al.*, 2014b; this study). In cultivated rice, allantoin also induced stress responses by significantly increasing the levels of osmoprotectants such as soluble sugars and free proline (Wang *et al.*, 2012). Although none of these lines of evidence is as yet conclusive, it is worth mentioning that these possible actions of allantoin are not mutually exclusive, which suggests that this purine metabolite may have a role beyond its function as a metabolic intermediate or nitrogen reservoir under stress conditions. Multifunctionality of small metabolites, in particular their signaling and regulatory roles, has recently gained attention as part of the set of adaptive mechanisms that allow plants to survive and grow under changing environments. The importance of various effects on plant development and physiology has been documented for certain molecular species of sugars, amino acids, polyamines, and other kinds of small compounds that are not considered to be phytohormones (see the Introduction). Currently, the number of such small metabolites is limited, but is estimated to be much higher than that reported in the literature (Tholl and Aharoni, 2014). Thus, it seems possible that allantoin could serve in either nitrogen metabolism or stress protection, depending on the circumstances where plants live. For further substantiation of such a dual functionality of allantoin, its modes of action in stress responses and adaptation need to be firmly defined.

## Supplementary data

Supplementary data are available at *JXB* online.

Methods S1. Quantification of JA and JA-Ile; accession numbers.

Figure S1. The purine catabolism pathway and metabolites derived therefrom.

Figure S2. Hierarchical tree graph of over-represented GO terms for genes with significantly increased expression in the *aln-1* mutant.

Figure S3. Hierarchical tree graph of over-represented GO terms for genes with significantly reduced expression in the *aln-1* mutant.

Figure S4. Basal level expression of *PR-1* as a canonical SA marker.

Figure S5. Characterization of the *aln-1 jar1-1* double mutant.

Figure S6. Reduced response to MeJA of anthocyanin accumulation in the *aah* mutant.

Figure S7. Characterization of the *aln-1 bglu18* double mutant.

Table S1. Primers used in this study.

Table S2. LC-ESI-MS/MS parameters for jasmonate determination.

Table S3. Genes with significantly increased expression in the *aln-1* mutant.

Table S4. Genes with significantly reduced expression in the *aln-1* mutant.

Supplementary Data
