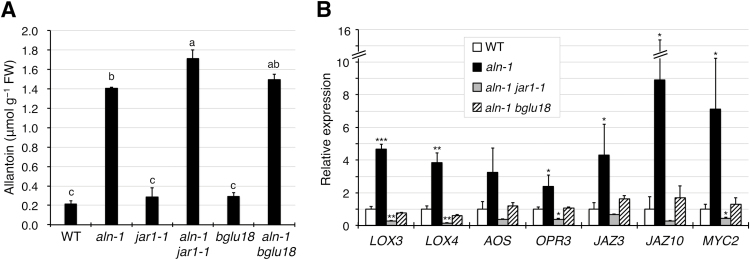# Allantoin, a stress-related purine metabolite, can activate jasmonate signaling in a MYC2-regulated and abscisic acid-dependent manner

**DOI:** 10.1093/jxb/erw289

**Published:** 2016-08-02

**Authors:** Hiroshi Takagi, Yasuhiro Ishiga, Shunsuke Watanabe, Tomokazu Konishi, Mayumi Egusa, Nobuhiro Akiyoshi, Takakazu Matsuura, Izumi C Mori, Takashi Hirayama, Hironori Kaminaka, Hiroshi Shimada, Atsushi Sakamoto

**Affiliations:** 1 Graduate School of Science, Hiroshima University, Higashi-Hiroshima, Japan; 2 Faculty of Life and Environmental Sciences, University of Tsukuba, Tsukuba, Japan; 3 Faculty of Bioresource Sciences, Akita Prefectural University, Akita, Japan; 4 Faculty of Agriculture, Tottori University, Tottori, Japan; 5 Institute of Plant Science and Resources, Okayama University, Kurashiki, Japan

*Journal of Experimental Botany*, Vol. 67, No. 8, pp. 2519–2532, 2016, doi: 10.1093/jxb/erw071.

In Figure 6B of the above paper, the real-time reverse-transcription quantitative PCR data for *JAZ3* were inadvertently used to represent the relative expression of both *JAZ3* and *MYC2*. The correct data for *MYC2* are provided in the revised version of this figure shown below. This correction does not affect the interpretation of the results or the conclusions of this work.

**Figure:**