# Supplementary material for: Allantoin, a stress-related purine metabolite, can activate jasmonate signaling in a MYC2-regulated and abscisic acid-dependent manner
Source: J Exp Bot. 2016 Mar 1;67(8):2519–32. doi: 10.1093/jxb/erw071 (PMC4809300; doi:10.1093/jxb/erw071)
Supplement: Supplementary Data [file supp_67_8_2519__index.html]

Allantoin, a stress-related purine metabolite, can activate jasmonate signaling in a MYC2-regulated and abscisic acid-dependent manner — Allantoin, a stress-related purine metabolite, can activate jasmonate signaling in a MYC2-regulated and abscisic acid-dependent manner — Allantoin, a stress-related purine metabolite, can activate jasmonate signaling in a MYC2-regulated and abscisic acid-dependent manner — Supplementary Data 

# Allantoin, a stress-related purine metabolite, can activate jasmonate signaling in a MYC2-regulated and abscisic acid-dependent manner

## Supplementary Data

Data files

- supplementary\_methods\_figures\_S1\_S7\_tables\_S1\_S4.pdf - Supplementary Data
