# Supplementary material for: Allantoin, a stress-related purine metabolite, can activate jasmonate signaling in a MYC2-regulated and abscisic acid-dependent manner
Source: J Exp Bot. 2016 Mar 1;67(8):2519–32. doi: 10.1093/jxb/erw071 (PMC4809300; doi:10.1093/jxb/erw071)
Supplement: Supplementary Data [file supp_erw071_supplementary_methods_figures_S1_S7_tables_S1_S4.pdf]

Supplementary Data

Allantoin, a stress-related purine metabolite, can activate jasmonate signaling in a MYC2-regulated and abscisic acid-dependent manner

Hiroshi Takagi, Yasuhiro Ishiga, Shunsuke Watanabe, Tomokazu Konishi, Mayumi Egusa, Nobuhiro Akiyoshi, Takakazu Matsuura, Izumi C. Mori, Takashi Hirayama, Hironori Kaminaka, Hiroshi Shimada, and Atsushi Sakamoto

The following Supplementary Data are available for this article:

Methods S1. Quantification of JA and JA-Ile; Accession numbers.

Figure S1. The purine catabolism pathway and metabolites derived therefrom.

Figure S2. Hierarchical tree graph of over-represented GO terms for genes with significantly increased expression in the *aln-1* mutant.

Figure S3. Hierarchical tree graph of over-represented GO terms for genes with significantly reduced expression in the *aln-1* mutant.

Figure S4. Basal level expression of *PR-1* as a canonical SA marker.

Figure S5. Characterization of the *aln-1 jar1-1* double mutant.

Figure S6. Reduced response to MeJA of anthocyanin accumulation in the *aah* mutant.

Figure S7. Characterization of the *aln-1 bglu18* double mutant.

Table S1. Primers used in this study.

Table S2. LC-ESI-MS/MS parameters for jasmonate determination.

Table S3. Genes with significantly increased expression in the *aln-1* mutant.

Table S4. Genes with significantly reduced expression in the *aln-1* mutant.

Supplementary Methods S1

Quantification of jasmonic acid (JA) and JA-Ile

Extraction and quantification of JA and JA-Ile were performed following the method of Preston *et al.* (2009) with minor modifications. The stable isotope-labeled compounds used as internal standards were: [$^{2}H_{2}$]JA (Tokyo Chemical industry Co., Ltd., Tokyo, Japan) and [$^{13}C_{6}$]JA-Ile, which was synthesized with [$^{13}C_{6}$]Ile (Cambridge Isotope Laboratories, Andover, MA, USA) as described in Jikumaru *et al.* (2004). Aerial parts (500 mg) from 2-week-old seedlings grown aseptically on solid half-strength MS medium were frozen in liquid N_2, ground, and extracted with 80% (v/v) methanol containing 1% (v/v) acetic acid at 4°C for 1 h. After removal of cell debris by centrifugation, the supernatants were condensed and dried *in vacuo*, and the resultant residues were extracted twice with methanol. The extracts were evaporated to dryness and resuspended with 80% methanol containing 1% acetic acid and internal standards, which were again evaporated and then extracted with water acidified with 1% (v/v) acetic acid. The extracts were loaded onto pre-equilibrated Oasis HLB column cartridge (Waters Corporation, Milford, MA, USA). After washed with water acidified with 1% acetic acid, the column was eluted with 80% (v/v) acetonitrile containing 1% (v/v) acetic acid. The eluted samples were evaporated to obtain extracts in water acidified with 1% acetic acid, and loaded onto a pre-equilibrated Oasis MCX column cartridge (Waters). The cartridge was washed with water acidified with 1% acetic acid and eluted with 80% acetonitrile containing 1% acetic acid. The eluate was loaded onto pre-equilibrated Oasis WAX column cartridges (Waters) followed by washing, first with water acidified 1% acetic acid and then with 80% acetonitrile, and the fraction containing JA and JA-Ile was eluted with 80% acetonitrile containing 1% acetic acid. The obtained fraction was dried and reconstituted in ultra-pure water acidified with 1% (v/v) acetic acid for quantification of JA and JA-Ile by liquid chromatography-electrospray ionization-tandem mass spectrometry (LC-ESI-MS/MS) on an Agilent 6410 Triple Quadrupole system with a ZORBAX Eclipse XDB-C18 column and MassHunter software version B.01.02 (Agilent Technology, Palo Alto, CA, USA). The LC column was eluted with a binary solvent system of 0.01% (v/v) acetic acid in water (solvent A) and 0.05% (v/v) acetic acid in acetonitrile (solvent B) using a linear gradient of solvent B in solvent A, from 3% to 50%, in 20 min at the flow rate of 0.4 ml min^{-1}. The MS/MS operation parameters are summarized in Supplementary Table S2.

Accession numbers

Arabidopsis Genome Initiative numbers for the genes mentioned in this article are as follows: *AAH*, At4g20070; *ABA2*, At1g52340; *ACT2*, At3g18780; *ALN*, At4g04955; *ANAC019*, At1g52890; *ANAC055*, At3g15500; *ANAC072*, At4g27410; *AOC1*, At3g25760; *AOS*, At5g42650; *BGLU18*, At1g52400; *BSMT1*, At3g11480; *CYP94B3*, At3g48520; *ERF1*, At3g23240; *ICS1*, At1g74710; *JAM1*, At2g46510; *JAR1*, At2g46370; *JAZ1*, At1g19180; *JAZ3*, At3g17860; *JAZ5*, At1g17380; *JAZ6*, At1g72450; *JAZ7*, At2g34600; *JAZ10*, At5g13220; *JAZ12*, At5g20900; *LOX2*, At3g45140; *LOX3*, At1g17420; *LOX4*, At1g72520; *MYC2*, At1g32640; *OPR3*, At2g06050; *ORA59*, At1g06160; *PDF1.2a*, At5g44420; *PDF1.2b*, At2g26020; *PR-1*, At2g14610; *SAGT1*, At2g43820; *VSP1*, At5g24780; and *XDH1*, At4g34890.

Supplementary Figure Legends

Figure S1. The purine catabolism pathway and metabolites derived therefrom.
The pathway is schematically illustrated starting from the common intermediate xanthine, at which all purine nucleotides converge when they are subjected to degradation. The abbreviated names of the genes encoding the enzymes that catalyze each step are shown in italics and knockout mutants used in this study are indicated in parentheses under the gene names: XDH, xanthine dehydrogenase; UOX, urate oxidase; AS, allantoin synthase; ALN, allantoinase (allantoin amidohydrolase); AAH, allantoate amidohydrolase; UGAH, ureidoglycine aminohydrolase; UAH, ureidoglycolate amidohydrolase.

Figure S2. Hierarchical tree graph of over-represented GO terms for genes with significantly increased expression in the *aln-1* mutant.
A total of 47 enriched GO terms under Biological Process were found for 211 genes with significantly increased expression (≥ 3-fold changes, Supplementary Table S3) using the BioMaps tool of VirtualPlant version 1.3 (Katari *et al*., 2010; http://virtualplant.bio.nyu.edu/cgi-bin/vpweb/) in the default-setting mode (Fisher's exact test with false discovery rate correction, $P < 0.01$). Each box shows the name of the GO term and, where appropriate, the P value (< 0.01) for the significance of enrichment. The P values are also indicated by the frame color of each box according to the gradient scale. The rank direction of the graph runs from top to bottom.

Figure S3. Hierarchical tree graph of over-represented GO terms for genes with significantly reduced expression in the *aln-1* mutant.
A total of 33 enriched GO terms under Biological Process were found for 113 genes with significantly reduced expression (≥ 3-fold changes, Supplementary Table S4) using the BioMaps tool of VirtualPlant version 1.3 (Katari *et al*., 2010; http://virtualplant.bio.nyu.edu/cgi-bin/vpweb/) in the default-setting mode (Fisher's exact test with false discovery rate correction, $P < 0.01$). Each box shows the name of the GO term and, where appropriate, the P value (< 0.01) for the significance of enrichment. The P values are also indicated by the frame color of each box according to the gradient scale. The rank direction of the graph runs from top to bottom.

Figure S4. Basal level expression of *PR-1* as a canonical SA marker.
RNA was extracted from aerial parts of 2-week-old seedlings of WT and *aln-1* mutants grown under normal aseptic conditions. Relative mRNA levels were determined by real-time reverse

transcription-quantitative PCR using *ACTIN2* expression as reference and presented as values relative to the WT level. The sequences of primers are listed in Supplementary Table S1. Data are means ± SEM from three independent experiments ($*P < 0.05$ by Student's *t*-test comparison to the WT level).

Figure S5. Characterization of the *aln-1 jar1-1* double mutant.
The homozygous mutants of *aln-1* (SALK_000325; Yang and Han, 2004; Watanabe *et al.*, 2014) and *jar1-1* (CS8072; Staswick *et al.*, 1992) were crossed to obtain the double mutant *aln-1 jar1-1*. (A) Diagram of the T-DNA insertion in the *ALN* gene in the *aln-1* mutant. Arrows denote PCR primers. (B) PCR-based genotyping of the double mutant using primers specific to *ALN* (F1 and R1) and the left border sequence of T-DNA (LBa1). (C) Diagram of the *JAR1* gene structure and the confirmation of the *jar1-1* mutation in the double mutant. The nucleotide sequence of the wild-type *JAR1* allele (*a*) from TAIR (At2g46370; https://www.arabidopsis.org/) was compared to that of the double mutant, as determined by dideoxy sequencing with primers F2 (*b*) and R2 (*c*), to confirm the single nucleotide missense mutation that occurs in exon 3 (Staswick *et al.*, 2002). The sequences of primers are listed in Supplementary Table S1. (D) Typical root growth of 8-day-old seedlings of WT, *jar1-1*, and *aln-1 jar1-1* genotypes in the presence of 10 μM methyl jasmonate (MeJA). Note that the root growth of the *jar1-1* mutant is moderately insensitive to MeJA (Staswick *et al.*, 1992, 2002). Horizontal bars indicate a scale of 10 mm in length (white) and the position of a primary root tip (yellow).

Figure S6. Reduced response to MeJA of anthocyanin accumulation in the *aah* mutant.
Sterile seedlings of WT and the *aah* mutant were grown for 8 days on standard medium supplemented with 10 μM MeJA and examined for anthocyanin accumulation as described in the main text. FW, fresh weight. Data are means ± SEM ($n = 8$; $*P < 0.001$ by Student's *t*-test comparison to the WT levels).

Figure S7. Characterization of the *aln-1 bglu18* double mutant.
The homozygous mutants of *aln-1* (SALK_000325; Yang and Han, 2004; Watanabe *et al.*, 2014) and *bglu18* (SALK_075731C; Ogasawara *et al.*, 2009) were crossed to obtain the double mutant *aln-1 bglu18*. (A) Diagram of the T-DNA insertion in the *BGLU18* gene in the *bglu18* mutant. Arrows denote PCR primers. (B) PCR-based genotyping of the double mutant using primers specific to *ALN* (F1 and R4; see Supplementary Fig. S5), *BGLU18* (F3 and R3), and the left border sequence of T-DNA (LBa1). The sequences of primers are listed in Supplementary Table S1.

Supplementary Table Legends

Table S1. Primers used in this study.

[a] *Arabidopsis thaliana* gene identifier code assigned by the Arabidopsis Genome Initiatives (AGI; https://www.arabidopsis.org/portals/nomenclature/).

[b] Gene symbol as provided by The Arabidopsis Information Resource (TAIR; release 10; https://www.arabidopsis.org/) except for T-DNA of *Agrobacterium tumefaciens*.

Table S2. LC-ESI-MS/MS parameters for jasmonate determination.

Table S3. Genes with significantly increased expression in the *aln-1* mutant.

Listed are those whose changes in transcript levels increased by equal to or greater than 3-fold, with the statistical significance level of 0.001 by a two-way analysis of variance (ANOVA). These genes were selected from the revised microarray data (Gene Expression Omnibus accession number GSE73841) that had been parametrically renormalized using the SuperNORM data service (Skylight Biotech Inc, Akita, Japan), according to the three-parameter lognormal distribution method (Konishi, 2004).

[a] *Arabidopsis thaliana* gene identifier code assigned by the Arabidopsis Genome Initiatives (AGI; https://www.arabidopsis.org/portals/nomenclature/).

[b] Mean of two independent biological experiments (*aln-1* versus wild-type).

[c] Gene symbol and description as provided by The Arabidopsis Information Resource (TAIR; release 10; https://www.arabidopsis.org/).

[d] *P*-value determined by a two-way ANOVA.

Table S4. Genes with significantly reduced expression in the *aln-1* mutant.

Listed are those whose changes in transcript levels decreased by equal to or greater than 3-fold with the statistical significance level of 0.001 by a two-way ANOVA. These genes were selected from the revised microarray data (Gene Expression Omnibus accession number GSE73841) that had been parametrically renormalized using the SuperNORM data service (Skylight Biotech Inc, Akita, Japan), according to the three-parameter lognormal distribution method (Konishi, 2004).

[a] *Arabidopsis thaliana* gene identifier code assigned by the Arabidopsis Genome Initiatives (AGI; https://www.arabidopsis.org/portals/nomenclature/).

[b] Mean of two independent biological experiments (*aln-1* versus wild-type).

[c] Gene symbol and description as provided by The Arabidopsis Information Resource (TAIR; release 10; https://www.arabidopsis.org/).

[d] *P*-value determined by a two-way ANOVA.

Supplementary References

Jikumaru Y, Asami T, Seto H, *et al.* 2004. Preparation and biological activity of molecular probes to identify and analyze jasmonic acid-binding proteins. Bioscience, Biotechnology, and Biochemistry **68,** 1461–1466.

Katari MS, Nowicki SD, Aceituno FF, *et al.* 2010. VirtualPlant: a software platform to support systems biology research. Plant Physiology **152**, 500–515.

Konishi T. 2004. Three-parameter lognormal distribution ubiquitously found in cDNA microarray data and its application to parametric data treatment. BMC Bioinformatics **5**, 5.

Ogasawara K, Yamada K, Christeller JT, Kondo M, Hatsugai N, Hara-Nishimura I, Nishimura M. 2009. Constitutive and inducible ER bodies of *Arabidopsis thaliana* accumulate distinct ß-glucosidases. Plant and Cell Physiology **50**, 480–488.

Preston J, Tatematsu K, Kanno Y, Hobo T, Kimura M, Jikumaru Y, Yano R, Kamiya Y, Nambara E. 2009. Temporal expression patterns of hormone metabolism genes during imbibition of *Arabidopsis thaliana* seeds: A comparative study on dormant and non-dormant accessions. Plant and Cell Physiology **50**, 1786–1800.

Staswick PE, Su W, Howell SH. 1992. Methyl jasmonate inhibition of root growth and induction of a leaf protein are decreased in an *Arabidopsis thaliana* mutant. Proceedings of the National Academy of Sciences of the United States of America **89**, 6837–6840.

Staswick PE, Tiryaki I, Rowe ML. 2002. Jasmonate response locus *JAR1* and several related Arabidopsis genes encode enzymes of the firefly luciferase superfamily that show activity on jasmonic, salicylic, and indole-3-acetic acids in an assay for adenylation. The Plant Cell **14**, 1405–1415.

Watanabe S, Matsumoto M, Hakomori Y, Takagi H, Shimada H, Sakamoto A. 2014. The purine metabolite allantoin enhances abiotic stress tolerance through synergistic activation of abscisic acid metabolism. Plant, Cell & Environment **37**, 1022–1036.

Yang J, Han K-H. 2004. Functional characterization of allantoinase genes from Arabidopsis and a nonureide-type legume black locust. Plant Physiology **134**, 1039–1049.

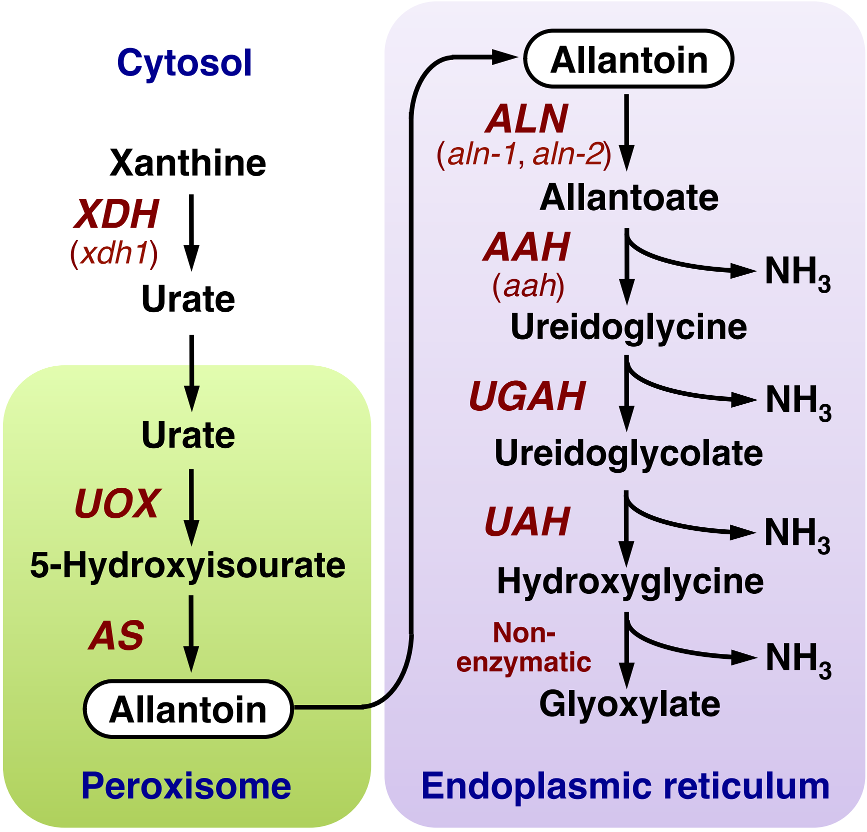

Figure S1. The purine catabolism pathway and metabolites derived therefrom.
The pathway is schematically illustrated starting from the common intermediate xanthine, at which all purine nucleotides converge when they are subjected to degradation. The abbreviated names of the genes encoding the enzymes that catalyze each step are shown in italics and knockout mutants used in this study are indicated in parentheses under the gene names: XDH, xanthine dehydrogenase; UOX, urate oxidase; AS, allantoin synthase; ALN, allantoinase (allantoin amidohydrolase); AAH, allantoate amidohydrolase; UGAH, ureidoglycine aminohydrolase; UAH, ureidoglycolate amidohydrolase.

Supplementary Figures

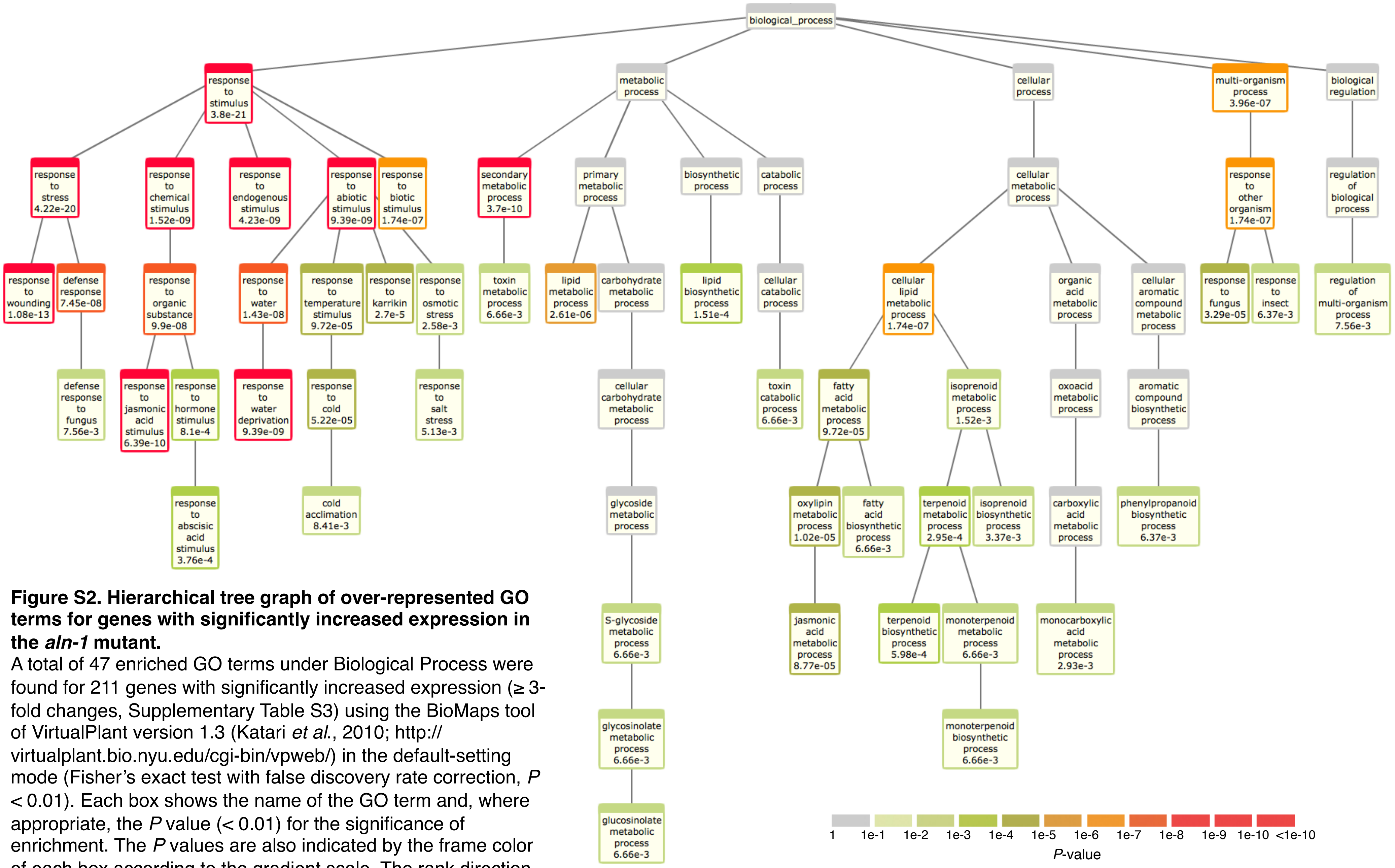

Figure S2. Hierarchical tree graph of over-represented GO terms for genes with significantly increased expression in the *aln-1* mutant.
A total of 47 enriched GO terms under Biological Process were found for 211 genes with significantly increased expression (≥ 3-fold changes, Supplementary Table S3) using the BioMaps tool of VirtualPlant version 1.3 (Katari *et al*., 2010; http://virtualplant.bio.nyu.edu/cgi-bin/vpweb/) in the default-setting mode (Fisher's exact test with false discovery rate correction, $P < 0.01$). Each box shows the name of the GO term and, where appropriate, the *P* value (< 0.01) for the significance of enrichment. The *P* values are also indicated by the frame color of each box according to the gradient scale. The rank direction of the graph runs from top to bottom.

Supplementary Figures

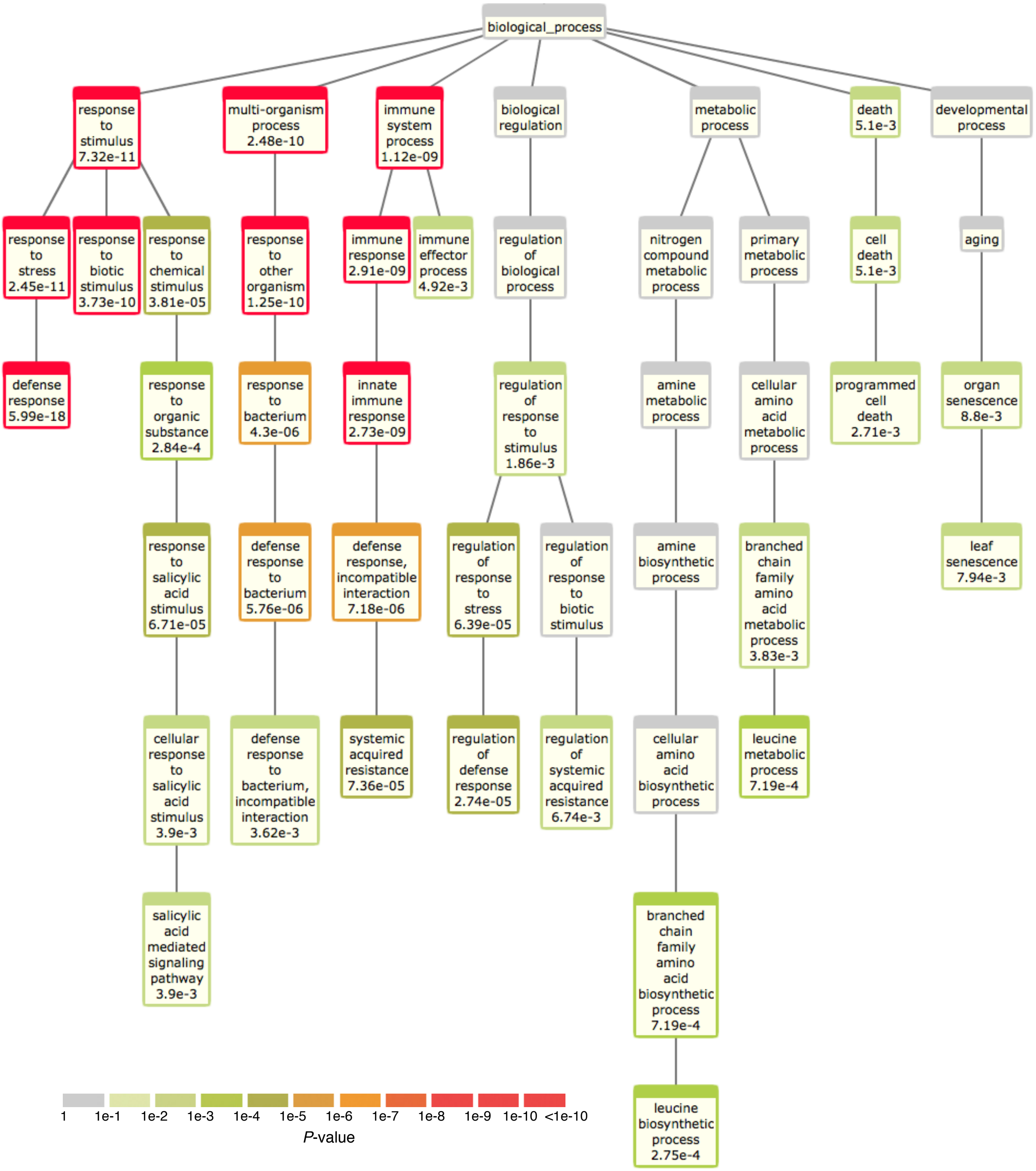

Figure S3. Hierarchical tree graph of over-represented GO terms for genes with significantly reduced expression in the *aln-1* mutant.

A total of 33 enriched GO terms under Biological Process were found for 113 genes with significantly reduced expression (≥ 3-fold changes, Supplementary Table S4) using the BioMaps tool of VirtualPlant version 1.3 (Katari *et al.*, 2010; http://virtualplant.bio.nyu.edu/cgi-bin/vpweb/) in the default-setting mode (Fisher's exact test with false discovery rate correction, $P < 0.01$). Each box shows the name of the GO term and, where appropriate, the P value (< 0.01) for the significance of enrichment. The P values are also indicated by the frame color of each box according to the gradient scale. The rank direction of the graph runs from top to bottom.

Supplementary Figures

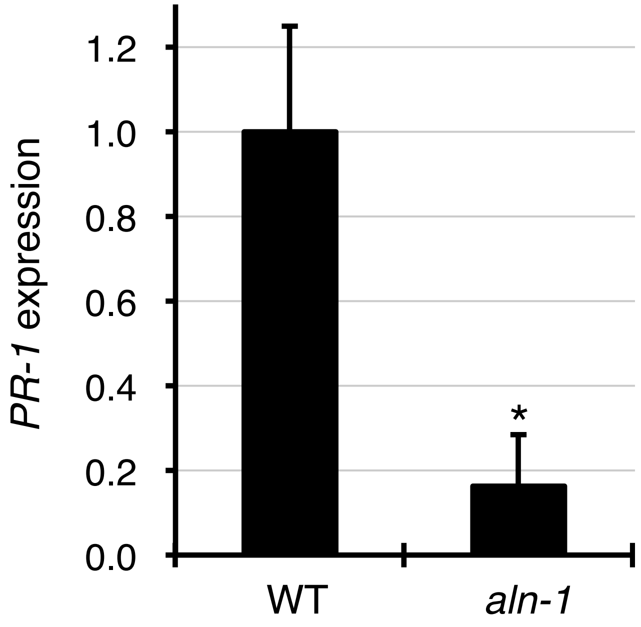

Figure S4. Basal level expression of *PR-1* as a canonical SA marker.
RNA was extracted from aerial parts of 2-week-old seedlings of WT and *aln-1* mutants grown under normal aseptic conditions. Relative mRNA levels were determined by real-time reverse transcription-quantitative PCR using *ACTIN2* expression as reference and presented as values relative to the WT level. The sequences of primers are listed in Supplementary Table S1. Data are means ± SEM from three independent experiments (*$P < 0.05$ by Student's *t*-test comparison to the WT level).

Supplementary Figures

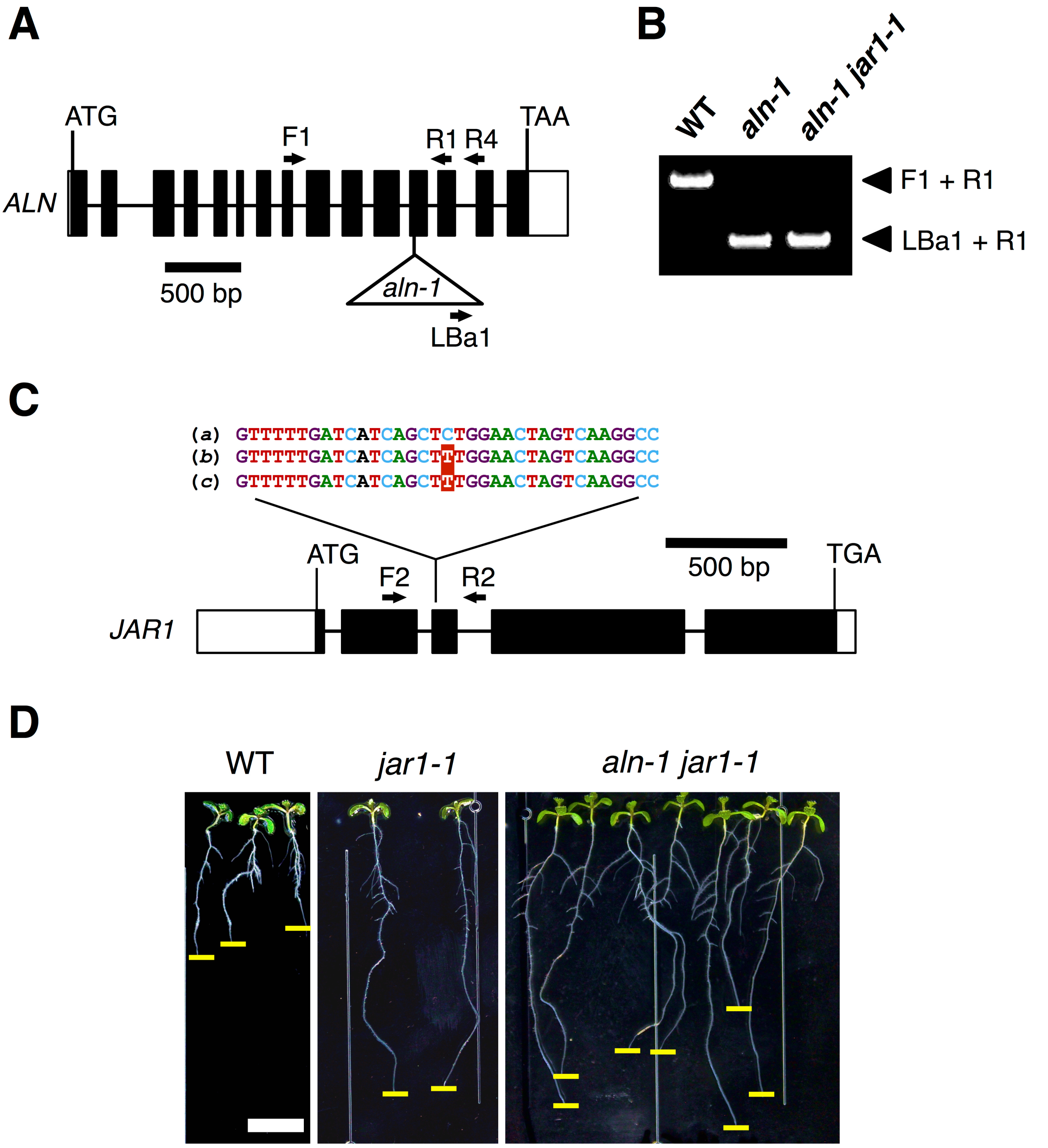

Figure S5. Characterization of the *aln-1 jar1-1* double mutant.
The homozygous mutants of *aln-1* (SALK_000325; Yang and Han, 2004; Watanabe *et al.*, 2014) and *jar1-1* (CS8072; Staswick *et al.*, 1992) were crossed to obtain the double mutant *aln-1 jar1-1*. (A) Diagram of the T-DNA insertion in the *ALN* gene in the *aln-1* mutant. Arrows denote PCR primers. (B) PCR-based genotyping of the double mutant using primers specific to *ALN* (F1 and R1) and the left border sequence of T-DNA (LBa1). (C) Diagram of the *JAR1* gene structure and the confirmation of the *jar1-1* mutation in the double mutant. The nucleotide sequence of the wild-type *JAR1* allele (*a*) from TAIR (At2g46370; https://www.arabidopsis.org/) was compared to that of the double mutant, as determined by dideoxy sequencing with primers F2 (*b*) and R2 (*c*), to confirm the single nucleotide missense mutation that occurs in exon 3 (Staswick *et al.*, 2002). The sequences of primers are listed in Supplementary Table S1. (D) Typical root growth of 8-day-old seedlings of WT, *jar1-1*, and *aln-1 jar1-1* genotypes in the presence of 10 µM methyl jasmonate (MeJA). Note that the root growth of the *jar1-1* mutant is moderately insensitive to MeJA (Staswick *et al.*, 1992, 2002). Horizontal bars indicate a scale of 10 mm in length (white) and the position of a primary root tip (yellow).

Supplementary Figures

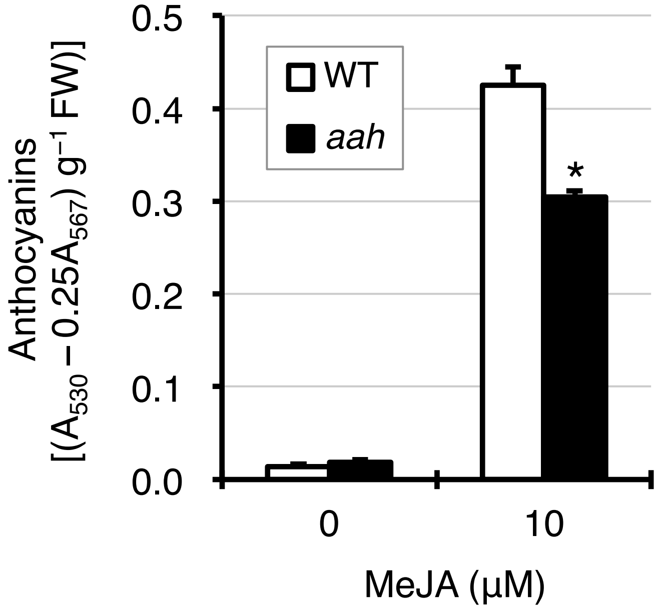

Figure S6. Reduced response to MeJA of anthocyanin accumulation in the *aah* mutant.
Sterile seedlings of WT and the *aah* mutant were grown for 8 days on standard medium supplemented with 10 µM MeJA and examined for anthocyanin accumulation as described in the main text. FW, fresh weight. Data are means ± SEM (n = 8; *P < 0.001 by Student's *t*-test comparison to the WT levels).

Supplementary Figures

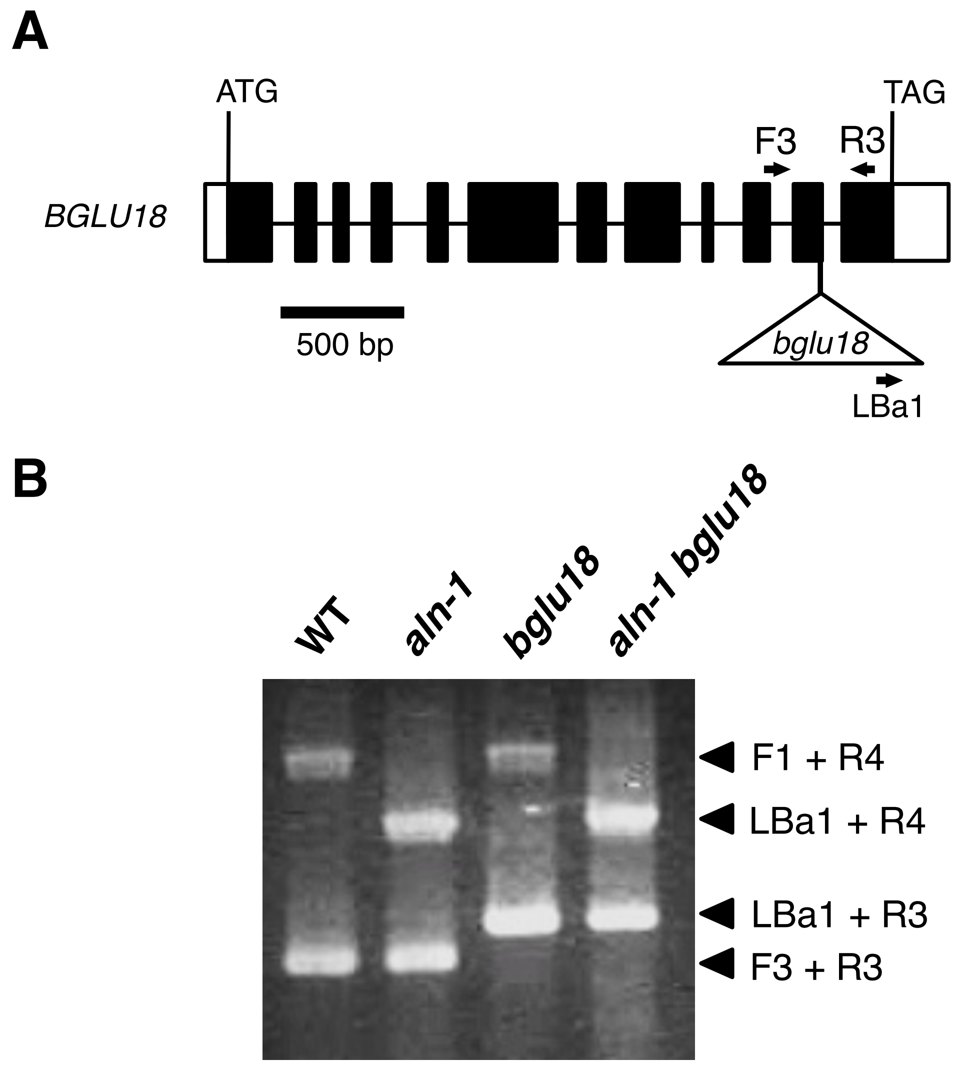

Figure S7. Characterization of the *aln-1 bglu18* double mutant.
The homozygous mutants of *aln-1* (SALK_000325; Yang and Han, 2004; Watanabe *et al.*, 2014) and *bglu18* (SALK_075731C; Ogasawara *et al.*, 2009) were crossed to obtain the double mutant *aln-1 bglu18*. (A) Diagram of the T-DNA insertion in the *BGLU18* gene in the *bglu18* mutant. Arrows denote PCR primers. (B) PCR-based genotyping of the double mutant using primers specific to *ALN* (F1 and R4; see Supplementary Fig. S5), *BGLU18* (F3 and R3), and the left border sequence of T-DNA (LBa1). The sequences of primers are listed in Supplementary Table S1.

Table S1. Primers used in this study

AGI[a]	Gene symbol[b]	Direction	Sequence (Designation)	Use
At4g04955	*ALN*	Forward	5'-CCTTTATGTGCCCTTCAGGA-3' (F1)	PCR genotyping
		Reverse	5'-GGCCTATCACTCCACCAAGA-3' (R1)	PCR genotyping
		Reverse	5'-GGTTCCCACACAACAAGATCTGC-3' (R4)	PCR genotyping
At1g52400	*BGLU18*	Forward	5'-GGCGACCCAGAAGTTATCAT-3' (F3)	PCR genotyping
		Reverse	5'-GAATACCATTTGCCCGAAAC-3' (R3)	PCR genotyping
At2g46370	*JAR1*	Forward	5'-CGCTACTGACCCTGAAGAAGCTTT-3' (F2)	DNA sequencing
		Reverse	5'-CAACATGTTAAGGCATAGTCG-3' (R2)	DNA sequencing
At1g17420	*LOX3*	Forward	5'-TGAACATTGAGAGAGTCAAGACTTTT-3'	RT-qPCR
		Reverse	5'-AGATAGTTCGAGTAGCATAGGCTTT-3'	RT-qPCR
At1g72520	*LOX4*	Forward	5'-TCGCTAACTTTGGTGAGATCGATAG-3'	RT-qPCR
		Reverse	5'-TCGTCATCTCGAAGCCATGCATATT-3'	RT-qPCR
At5g42650	*AOS*	Forward	5'-GGTGGCGAGGTTGTTTGTGA-3'	RT-qPCR
		Reverse	5'-GCGACGTACCAACCTCAATATCA-3'	RT-qPCR
At2g06050	*OPR3*	Forward	5'-ACGGACCACTCCCGGCGGTTTT-3'	RT-qPCR
		Reverse	5'-CGTGAACTGCTTCCACAACT-3'	RT-qPCR
At1g32640	*MYC2*	Forward	5'-CGCGAGTATGTCGGTGGTTA-3'	RT-qPCR
		Reverse	5'-TGCTCTGAGCTGTTCTTGCGTATA-3'	RT-qPCR
At3g17860	*JAZ3*	Forward	5'-GTTCTACCAATGTAATGGCTCCAACA-3'	RT-qPCR
		Reverse	5'-CAATATGGGGATACGCTCGT-3'	RT-qPCR
At5g13220	*JAZ10*	Forward	5'-AGCTCTTTGGCCAGAATCTAGA-3'	RT-qPCR
		Reverse	5'-AGATGTTGATACTAATCTCTCCTTG-3'	RT-qPCR
At2g14610	*PR-1*	Forward	5'-CGTCTTTGTAGCTCTTGTAGGTGC-3'	RT-qPCR
		Reverse	5'-TGCCTGGTTGTGAACCCTTAG-3'	RT-qPCR
At5g44420	*PDF1.2a*	Forward	5'-CTTGTTCTCTTTGCTGCTTTCGAC-3'	RT-qPCR
		Reverse	5'-TTGGCTCCTTCAAGGTTAATGCAC-3'	RT-qPCR
At3g23240	*ERF1*	Forward	5'-CGATCCCTAACCGAAAACAG-3'	RT-qPCR
		Reverse	5'-TCCGATAGAATATTCCGGTGA-3'	RT-qPCR
At3g18780	*ACTIN2*	Forward	5'-ACCGTATGAGCAAAGAAATCAC-3'	RT-qPCR
		Reverse	5'-GAGGGAAGCAAGAATGGAAC-3'	RT-qPCR
–	T-DNA	–	5'-TGGTTCACGTAGTGGGCCATCG-3' (LBa1)	PCR genotyping

[a] *Arabidopsis thaliana* gene identifier code assigned by the Arabidopsis Genome Initiatives (AGI; https://www.arabidopsis.org/portals/nomenclature/).

[b] Gene symbol as provided by The Arabidopsis Information Resource (TAIR; release 10; https://www.arabidopsis.org/) except for T-DNA of *Agrobacterium tumefaciens*.

Table S2. LC-ESI-MS/MS parameters for jasmonate determination

Analyte	Retention time on LC (min)	ESI mode	$[M–H]^{-1}$ (*m*/*z*)	Transition ion (*m*/*z*)	Collision energy (eV)	Fragmentor voltage (V)
JA	14.4	negative	209	59	15	135
$[^{2}H_2]$JA	14.4	negative	211	59	15	135
JA-Ile	18.0	negative	322.0	130	14	140
$[^{13}C_6]$JA-Ile	18.0	negative	328.4	136.2	14	140

Table S3. Genes with significantly increased expression in the *aln-1* mutant.

Listed are those whose changes in transcript levels increased by equal to or greater than 3-fold, with the statistical significance level of 0.001 by a two-way analysis of variance (ANOVA). These genes were selected from the revised microarray data (Gene Expression Omnibus accession number GSE73841) that had been parametrically renormalized using the SuperNORM data service (Skylight Biotech Inc, Akita, Japan), according to the three-parameter lognormal distribution method (Konishi 2004).

Affymetrix probe ID	AGI[a]	Fold change[b]	Gene symbol and description[c]	*P*-value[d]
248727_at	At5g47990	37.17	CYP705A5, THAD, THAD1, cytochrome P450, family 705, subfamily A, polypeptide 5	1.331E-06
252368_at	At3g48520	30.04	CYP94B3, cytochrome P450, family 94, subfamily B, polypeptide 3	1.282E-08
256601_s_at	At3g28290	18.06	AT14A, Protein of unknown function (DUF677)	1.411E-22
264415_at	At1g43160	16.76	RAP2.6, related to AP2 6	1.831E-14
267147_at	At2g38240	16.69	2-oxoglutarate (2OG) and Fe(II)-dependent oxygenase superfamily protein	3.205E-09
246340_s_at	At3g44860	14.61	FAMT, farnesoic acid carboxyl-O-methyltransferase	1.956E-21
249812_at	At5g23830	13.24	MD-2-related lipid recognition domain-containing protein	3.712E-16
263023_at	At1g23960	12.60	Protein of unknown function (DUF626)	6.221E-08
255065_s_at	At4g08870	12.06	Arginase/deacetylase superfamily protein	2.889E-26
265119_at	At1g62570	11.83	FMO GS-OX4, flavin-monooxygenase glucosinolate S-oxygenase 4	6.567E-13
248944_at	At5g45500	11.47	RNI-like superfamily protein	2.861E-06
247360_at	At5g63450	11.44	CYP94B1, cytochrome P450, family 94, subfamily B, polypeptide 1	5.902E-14
255257_at	At4g05050	10.42	UBQ11, ubiquitin 11	2.298E-25
261804_at	At1g30530	10.36	UGT78D1, UDP-glucosyl transferase 78D1	1.783E-21
266271_at	At2g29440	9.53	ATGSTU6, GST24, GSTU6, glutathione S-transferase tau 6	1.447E-09
267261_at	At2g23120	9.26	Late embryogenesis abundant protein, group 6	2.610E-14
256497_at	At1g31580	9.03	CXC750, ECS1, ECS1	1.596E-08
249600_s_at	At5g38000	8.90	Zinc-binding dehydrogenase family protein	5.939E-04
252487_at	At3g46660	8.73	UGT76E12, UDP-glucosyl transferase 76E12	4.360E-12
254481_at	At4g20480	8.62	Putative endonuclease or glycosyl hydrolase	6.565E-10
247175_at	At5g65280	8.21	GCL1, GCR2-like 1	1.076E-13
266901_at	At2g34600	7.87	JAZ7, TIFY5B, jasmonate-zim-domain protein 7	4.125E-16
263673_at	At2g04800	7.75	unknown protein; FUNCTIONS IN: molecular_function unknown; INVOLVED IN: N-terminal protein myristoylation; LOCATED IN: cellular_component unknown; EXPRESSED IN: hypocotyl, root; Has 4 Blast hits to 4 proteins in 1 species: Archae - 0; Bacteria - 0; Metazoa - 0; Fungi - 0; Plants - 4; Viruses - 0; Other Eukaryotes - 0 (source: NCBI BLink).	3.573E-05
256114_at	At1g16850	7.50	unknown protein; FUNCTIONS IN: molecular_function unknown; INVOLVED IN: response to salt stress; LOCATED IN: endomembrane system; EXPRESSED IN: leaf apex, leaf whorl, male gametophyte, flower, leaf; EXPRESSED DURING: LP.06 six leaves visible, LP.04 four leaves visible, LP.10 ten leaves visible, petal differentiation and expansion stage, LP.08 eight leaves visible; BEST Arabidopsis thaliana protein match is: unknown protein (TAIR:AT5G64820.1); Has 24 Blast hits to 24 proteins in 6 species: Archae - 0; Bacteria - 0; Metazoa - 0; Fungi - 0; Plants - 24; Viruses - 0; Other Eukaryotes - 0 (source: NCBI BLink).	6.812E-24
249599_at	At5g37990	7.10	S-adenosyl-L-methionine-dependent methyltransferases superfamily protein	2.658E-11
253104_at	At4g36010	7.06	Pathogenesis-related thaumatin superfamily protein	1.523E-16
263320_at	At2g47180	7.02	AtGolS1, GolS1, galactinol synthase 1	4.882E-18
249567_at	At5g38020	6.81	S-adenosyl-L-methionine-dependent methyltransferases superfamily protein	7.348E-16
263594_at	At2g01880	6.70	ATPAP7, PAP7, purple acid phosphatase 7	5.248E-11
260744_at	At1g15010	6.68	unknown protein; BEST Arabidopsis thaliana protein match is: unknown protein (TAIR:AT2G01300.1); Has 71 Blast hits to 71 proteins in 13 species: Archae - 0; Bacteria - 2; Metazoa - 0; Fungi - 0; Plants - 69; Viruses - 0; Other Eukaryotes - 0 (source: NCBI BLink).	7.417E-08
245689_at	At5g04120	6.23	Phosphoglycerate mutase family protein	4.586E-14
248205_at	At5g54300	6.15	Protein of unknown function (DUF761)	9.230E-12
265615_at	At2g25450	6.03	2-oxoglutarate (2OG) and Fe(II)-dependent oxygenase superfamily protein	1.288E-09
251513_at	At3g59220	5.98	ATPIRIN1, PRN, PRN1, pirin	2.923E-25
245444_at	At4g16740	5.97	ATTPS03, TPS03, terpene synthase 03	1.499E-11
246825_at	At5g26260	5.91	TRAF-like family protein	9.195E-12
247136_at	At5g66170	5.87	STR18, sulfurtransferase 18	4.901E-16
259286_at	At3g11480	5.84	ATBSMT1, BSMT1, S-adenosyl-L-methionine-dependent methyltransferases superfamily protein	2.060E-24
260549_at	At2g43535	5.84	Scorpion toxin-like knottin superfamily protein	4.788E-13
263482_at	At2g03980	5.82	GDSL-like Lipase/Acylhydrolase superfamily protein	1.014E-25
248392_at	At5g52050	5.71	MATE efflux family protein	1.438E-09
245555_at	At4g15390	5.70	HXXXD-type acyl-transferase family protein	4.897E-16
266743_at	At2g02990	5.64	ATRNS1, RNS1, ribonuclease 1	5.145E-10
265530_at	At2g06050	5.59	DDE1, OPR3, oxophytodienoate-reductase 3	2.070E-17
250793_at	At5g05600	5.56	2-oxoglutarate (2OG) and Fe(II)-dependent oxygenase superfamily protein	5.826E-15
251356_at	At3g61060	5.53	AtPP2-A13, PP2-A13, phloem protein 2-A13	2.022E-16
246488_at	At5g16010	5.52	3-oxo-5-alpha-steroid 4-dehydrogenase family protein	2.942E-12
245928_s_at	At5g24780	5.47	ATVSP1, VSP1, vegetative storage protein 1	3.363E-22
247600_at	At5g60890	5.40	ATMYB34, ATR1, MYB34, myb domain protein 34	9.715E-13
266246_at	At2g27690	5.38	CYP94C1, cytochrome P450, family 94, subfamily C, polypeptide 1	1.253E-12
252102_at	At3g50970	5.37	LTI30, XERO2, dehydrin family protein	4.219E-11
251241_s_at	At3g62460	5.37	Putative endonuclease or glycosyl hydrolase	9.894E-20
245713_at	At5g04370	5.34	NAMT1, S-adenosyl-L-methionine-dependent methyltransferases superfamily protein	1.362E-06
267411_at	At2g34930	5.34	disease resistance family protein / LRR family protein	4.288E-11
263083_at	At2g27190	5.31	ATPAP1, ATPAP12, PAP1, PAP12, purple acid phosphatase 12	6.013E-21
245749_at	At1g51090	5.29	Heavy metal transport/detoxification superfamily protein	6.242E-13
250662_at	At5g07010	5.22	ATST2A, ST2A, sulfotransferase 2A	1.799E-06
264886_at	At1g61120	5.22	GES, TPS04, TPS4, terpene synthase 04	1.201E-08
259846_at	At1g72140	5.22	Major facilitator superfamily protein	2.148E-11

261919_at	At1g65980	5.21	TPX1, thioredoxin-dependent peroxidase 1	4.164E-11
261037_at	At1g17420	5.19	LOX3, lipoxygenase 3	1.737E-22
248337_at	At5g52310	5.11	COR78, LTI140, LTI78, RD29A, low-temperature-responsive protein 78 (LTI78) / desiccation-responsive protein 29A (RD29A)	1.912E-13
257638_at	At3g25820	5.06	ATTPS-CIN, TPS-CIN, terpene synthase-like sequence-1,8-cineole	3.374E-19
266799_at	At2g22860	5.06	ATPSK2, PSK2, phytosulfokine 2 precursor	3.091E-09
262226_at	At1g53885	4.94	Protein of unknown function (DUF581)	4.589E-07
249971_at	At5g19110	4.92	Eukaryotic aspartyl protease family protein	3.110E-16
256324_at	At1g66760	4.87	MATE efflux family protein	1.026E-06
249101_at	At5g43580	4.74	Serine protease inhibitor, potato inhibitor I-type family protein	1.325E-06
250292_at	At5g13220	4.73	JAS1, JAZ10, TIFY9, jasmonate-zim-domain protein 10	5.410E-15
255527_at	At4g02360	4.67	Protein of unknown function, DUF538	5.697E-16
260969_at	At1g12240	4.64	ATBETAFRUCT4, VAC-INV, Glycosyl hydrolases family 32 protein	1.631E-20
255132_at	At4g08170	4.61	Inositol 1,3,4-trisphosphate 5/6-kinase family protein	9.523E-16
264146_at	At1g02205	4.58	CER1, Fatty acid hydroxylase superfamily	1.358E-10
252377_at	At3g47960	4.58	Major facilitator superfamily protein	9.941E-12
251023_at	At5g02170	4.56	Transmembrane amino acid transporter family protein	2.740E-06
260012_at	At1g67865	4.56	unknown protein; BEST Arabidopsis thaliana protein match is: unknown protein (TAIR:AT1G67860.1); Has 13 Blast hits to 13 proteins in 2 species: Archae - 0; Bacteria - 0; Metazoa - 0; Fungi - 0; Plants - 13; Viruses - 0; Other Eukaryotes - 0 (source: NCBI BLink).	1.160E-12
266800_at	At2g22880	4.53	VQ motif-containing protein	2.547E-09
254085_at	At4g24960	4.51	ATHVA22D, HVA22D, HVA22 homologue D	1.755E-15
264953_at	At1g77120	4.49	ADH, ADH1, ATADH, ATADH1, alcohol dehydrogenase 1	2.059E-15
250648_at	At5g06760	4.45	LEA4-5, Late Embryogenesis Abundant 4-5	1.309E-13
254326_at	At4g22610	4.45	Bifunctional inhibitor/lipid-transfer protein/seed storage 2S albumin superfamily protein	5.600E-10
265698_at	At2g32160	4.30	S-adenosyl-L-methionine-dependent methyltransferases superfamily protein	1.780E-07
247933_at	At5g56980	4.29	unknown protein; FUNCTIONS IN: molecular_function unknown; INVOLVED IN: biological_process unknown; LOCATED IN: endomembrane system; EXPRESSED IN: 18 plant structures; EXPRESSED DURING: 12 growth stages; BEST Arabidopsis thaliana protein match is: unknown protein (TAIR:AT4G26130.1); Has 30201 Blast hits to 17322 proteins in 780 species: Archae - 12; Bacteria - 1396; Metazoa - 17338; Fungi - 3422; Plants - 5037; Viruses - 0; Other Eukaryotes - 2996 (source: NCBI BLink).	9.334E-12
256417_s_at	At3g11170	4.27	FAD7, FADD, fatty acid desaturase 7	9.180E-14
245449_at	At4g16870	4.25	transposable element gene	1.839E-09
260399_at	At1g72520	4.20	PLAT/LH2 domain-containing lipoxygenase family protein	6.363E-16
258893_at	At3g05660	4.18	AtRLP33, RLP33, receptor like protein 33	4.862E-12
262496_at	At1g21790	4.17	TRAM, LAG1 and CLN8 (TLC) lipid-sensing domain containing protein	2.605E-19
264527_at	At1g30760	4.17	FAD-binding Berberine family protein	2.771E-08
251984_at	At3g53260	4.16	ATPAL2, PAL2, phenylalanine ammonia-lyase 2	2.247E-12
255942_at	At1g22360	4.12	AtUGT85A2, UGT85A2, UDP-glucosyl transferase 85A2	7.535E-13
248978_at	At5g45070	4.09	AtPP2-A8, PP2-A8, phloem protein 2-A8	7.511E-05
266486_at	At2g47950	4.07	unknown protein; FUNCTIONS IN: molecular_function unknown; INVOLVED IN: biological_process unknown; LOCATED IN: endomembrane system; EXPRESSED IN: root, flower; EXPRESSED DURING: petal differentiation and expansion stage; BEST Arabidopsis thaliana protein match is: unknown protein (TAIR:AT3G62990.1); Has 22 Blast hits to 22 proteins in 5 species: Archae - 0; Bacteria - 0; Metazoa - 0; Fungi - 0; Plants - 22; Viruses - 0; Other Eukaryotes - 0 (source: NCBI BLink).	2.353E-07
262010_at	At1g35612	4.07	transposable element gene	5.905E-06
263161_at	At1g54020	4.06	GDSL-like Lipase/Acylhydrolase superfamily protein	1.533E-12
245628_at	At1g56650	4.05	ATMYB75, MYB75, PAP1, SIAA1, production of anthocyanin pigment 1	2.806E-08
260557_at	At2g43610	4.04	Chitinase family protein	6.695E-19
258941_at	At3g09940	4.03	ATMDAR3, MDAR2, MDAR3, MDHAR, monodehydroascorbate reductase	1.933E-08
246125_at	At5g19875	4.03	unknown protein; FUNCTIONS IN: molecular_function unknown; INVOLVED IN: response to oxidative stress; LOCATED IN: endomembrane system; EXPRESSED IN: 21 plant structures; EXPRESSED DURING: 13 growth stages; BEST Arabidopsis thaliana protein match is: unknown protein (TAIR:AT2G31940.1); Has 30201 Blast hits to 17322 proteins in 780 species: Archae - 12; Bacteria - 1396; Metazoa - 17338; Fungi - 3422; Plants - 5037; Viruses - 0; Other Eukaryotes - 2996 (source: NCBI BLink).	3.758E-16
245736_at	At1g73330	4.03	ATDR4, DR4, drought-repressed 4	8.670E-15
248293_at	At5g53050	3.98	alpha/beta-Hydrolases superfamily protein	5.520E-14
258181_at	At3g21670	3.95	Major facilitator superfamily protein	9.060E-16
254561_at	At4g19160	3.95	unknown protein; Has 30201 Blast hits to 17322 proteins in 780 species: Archae - 12; Bacteria - 1396; Metazoa - 17338; Fungi - 3422; Plants - 5037; Viruses - 0; Other Eukaryotes - 2996 (source: NCBI BLink).	4.712E-21
245651_s_at	At1g24793	3.92	UDP-3-O-acyl N-acetylglycosamine deacetylase family protein	3.960E-25
267206_at	At2g30830	3.92	2-oxoglutarate (2OG) and Fe(II)-dependent oxygenase superfamily protein	5.079E-05
262637_at	At1g06640	3.91	2-oxoglutarate (2OG) and Fe(II)-dependent oxygenase superfamily protein	2.945E-14
258421_at	At3g16690	3.90	Nodulin MtN3 family protein	1.718E-06
258321_at	At3g22840	3.89	ELIP, ELIP1, Chlorophyll A-B binding family protein	9.380E-09
262887_at	At1g14780	3.88	MAC/Perforin domain-containing protein	5.670E-07
249216_at	At5g42240	3.88	scpl42, serine carboxypeptidase-like 42	5.393E-09
246286_at	At1g31910	3.88	GHMP kinase family protein	1.332E-22
254667_at	At4g18280	3.88	glycine-rich cell wall protein-related	2.040E-15
252170_at	At3g50480	3.86	HR4, homolog of RPW8 4	1.208E-10
255403_at	At4g03400	3.84	DFL2, GH3-10, Auxin-responsive GH3 family protein	9.628E-16
245244_at	At1g44350	3.83	ILL6, IAA-leucine resistant (ILR)-like gene 6	4.611E-14
264424_at	At1g61740	3.83	Sulfite exporter TauE/SafE family protein	3.458E-17
253259_at	At4g34410	3.81	RRTF1, redox responsive transcription factor 1	1.297E-05

248079_at	At5g55790	3.80	unknown protein; BEST Arabidopsis thaliana protein match is: unknown protein (TAIR:AT1G45163.1); Has 1807 Blast hits to 1807 proteins in 277 species: Archae - 0; Bacteria - 0; Metazoa - 736; Fungi - 347; Plants - 385; Viruses - 0; Other Eukaryotes - 339 (source: NCBI BLink).	9.744E-06
246987_at	At5g67300	3.79	ATMYB44, ATMYBR1, MYB44, MYBR1, myb domain protein r1	2.831E-14
267058_at	At2g32510	3.78	MAPKKK17, mitogen-activated protein kinase kinase kinase 17	4.132E-15
256994_s_at	At3g25830	3.77	ATTPS-CIN, TPS-CIN, TPS-CIN, terpene synthase-like sequence-1,8-cineole	1.801E-16
264787_at	At2g17840	3.76	ERD7, Senescence/dehydration-associated protein-related	7.595E-15
249010_at	At5g44580	3.73	unknown protein; FUNCTIONS IN: molecular_function unknown; INVOLVED IN: biological_process unknown; LOCATED IN: endomembrane system; EXPRESSED IN: 18 plant structures; EXPRESSED DURING: 12 growth stages; BEST Arabidopsis thaliana protein match is: unknown protein (TAIR:AT5G44582.1); Has 30201 Blast hits to 17322 proteins in 780 species: Archae - 12; Bacteria - 1396; Metazoa - 17338; Fungi - 3422; Plants - 5037; Viruses - 0; Other Eukaryotes - 2996 (source: NCBI BLink).	1.500E-18
245450_at	At4g16880	3.72	Leucine-rich repeat (LRR) family protein	2.548E-06
253814_at	At4g28290	3.69	unknown protein; FUNCTIONS IN: molecular_function unknown; INVOLVED IN: biological_process unknown; LOCATED IN: chloroplast; EXPRESSED IN: 23 plant structures; EXPRESSED DURING: 15 growth stages; Has 45 Blast hits to 45 proteins in 11 species: Archae - 0; Bacteria - 0; Metazoa - 0; Fungi - 0; Plants - 45; Viruses - 0; Other Eukaryotes - 0 (source: NCBI BLink).	7.458E-08
247982_at	At5g56760	3.69	ATSERAT1;1, SAT-52, SAT5, SERAT1;1, serine acetyltransferase 1;1	2.241E-15
248522_at	At5g50565	3.69	unknown protein; BEST Arabidopsis thaliana protein match is: unknown protein (TAIR:AT5G50665.2); Has 6 Blast hits to 6 proteins in 1 species: Archae - 0; Bacteria - 0; Metazoa - 0; Fungi - 0; Plants - 6; Viruses - 0; Other Eukaryotes - 0 (source: NCBI BLink).	7.287E-08
246432_at	At5g17490	3.68	RGL3, RGA-like protein 3	2.466E-09
251642_at	At3g57520	3.67	AtSIP2, SIP2, seed imbibition 2	1.617E-12
256725_at	At2g34070	3.67	TBL37, TRICHOME BIREFRINGENCE-LIKE 37	2.501E-15
263098_at	At2g16005	3.67	MD-2-related lipid recognition domain-containing protein	9.852E-09
262517_at	At1g17180	3.66	ATGSTU25, GSTU25, glutathione S-transferase TAU 25	3.372E-05
259802_at	At1g72260	3.65	THI2.1, THI2.1.1, thionin 2.1	1.612E-13
254306_at	At4g22330	3.64	ATCES1, Alkaline phytoceramidase (aPHC)	7.765E-14
248790_at	At5g47450	3.63	ATTIP2;3, DELTA-TIP3, TIP2;3, tonoplast intrinsic protein 2;3	7.061E-13
263497_at	At2g42540	3.61	COR15, COR15A, cold-regulated 15a	6.977E-23
252563_at	At3g45970	3.60	ATEXLA1, ATEXPL1, ATHEXP BETA 2.1, EXLA1, EXPL1, expansin-like A1	3.517E-14
247478_at	At5g62360	3.58	Plant invertase/pectin methylesterase inhibitor superfamily protein	5.862E-08
257952_at	At3g21770	3.56	Peroxidase superfamily protein	4.333E-12
264835_at	At1g03550	3.56	Secretory carrier membrane protein (SCAMP) family protein	7.277E-17
251012_at	At5g02580	3.55	Plant protein 1589 of unknown function	4.499E-06
259442_at	At1g02310	3.53	MAN1, Glycosyl hydrolase superfamily protein	4.281E-12
259758_s_at	At1g77530	3.52	O-methyltransferase family protein	2.696E-10
251229_at	At3g62740	3.51	BGLU7, beta glucosidase 7	1.563E-04
256577_at	At3g28220	3.51	TRAF-like family protein	2.151E-09
261199_at	At1g12950	3.46	RSH2, root hair specific 2	4.890E-06
245734_at	At1g73480	3.46	alpha/beta-Hydrolases superfamily protein	1.015E-15
264824_at	At1g03420	3.46	Sadhu4-2, transposable element gene	4.057E-11
259516_at	At1g20450	3.45	ERD10, LTI29, LTI45, Dehydrin family protein	1.119E-09
250161_at	At5g15240	3.45	Transmembrane amino acid transporter family protein	4.298E-07
261033_at	At1g17380	3.41	JAZ5, TIFY11A, jasmonate-zim-domain protein 5	1.707E-15
262516_at	At1g17190	3.41	ATGSTU26, GSTU26, glutathione S-transferase tau 26	1.011E-08
251028_at	At5g02230	3.41	Haloacid dehalogenase-like hydrolase (HAD) superfamily protein	2.489E-11
247431_at	At5g62520	3.40	SRO5, similar to RCD one 5	1.320E-04
257497_at	At1g51430	3.38	unknown protein; BEST Arabidopsis thaliana protein match is: unknown protein (TAIR:AT3G28370.1); Has 13 Blast hits to 13 proteins in 4 species: Archae - 0; Bacteria - 0; Metazoa - 0; Fungi - 0; Plants - 13; Viruses - 0; Other Eukaryotes - 0 (source: NCBI BLink).	7.165E-05
254809_at	At4g12410	3.37	SAUR-like auxin-responsive protein family	2.623E-17
254447_at	At4g20860	3.36	FAD-binding Berberine family protein	1.349E-04
256589_at	At3g28740	3.34	CYP81D1, Cytochrome P450 superfamily protein	3.269E-17
253832_at	At4g27654	3.34	unknown protein; FUNCTIONS IN: molecular_function unknown; INVOLVED IN: biological_process unknown; LOCATED IN: endomembrane system; EXPRESSED IN: 17 plant structures; EXPRESSED DURING: 9 growth stages; Has 30201 Blast hits to 17322 proteins in 780 species: Archae - 12; Bacteria - 1396; Metazoa - 17338; Fungi - 3422; Plants - 5037; Viruses - 0; Other Eukaryotes - 2996 (source: NCBI BLink).	1.072E-05
260357_at	At1g69260	3.32	AFP1, ABI five binding protein	6.224E-21
255723_at	At3g29575	3.32	AFP3, ABI five binding protein 3	5.297E-23
258498_at	At3g02480	3.30	Late embryogenesis abundant protein (LEA) family protein	1.938E-07
266832_at	At2g30040	3.29	MAPKKK14, mitogen-activated protein kinase kinase kinase 14	2.429E-11
258901_at	At3g05640	3.28	Protein phosphatase 2C family protein	6.072E-21
254909_at	At4g11210	3.27	Disease resistance-responsive (dirigent-like protein) family protein	2.215E-08
252677_at	At3g44320	3.26	AtNIT3, NIT3, nitrilase 3	5.307E-13
253827_at	At4g28085	3.26	unknown protein; Has 30201 Blast hits to 17322 proteins in 780 species: Archae - 12; Bacteria - 1396; Metazoa - 17338; Fungi - 3422; Plants - 5037; Viruses - 0; Other Eukaryotes - 2996 (source: NCBI BLink).	8.830E-13
264949_at	At1g77080	3.25	AGL27, FLM, MAF1, K-box region and MADS-box transcription factor family protein	1.637E-04
248524_s_at	At5g50570	3.24	Squamosa promoter-binding protein-like (SBP domain) transcription factor family protein	7.795E-20
262912_at	At1g59740	3.23	Major facilitator superfamily protein	2.947E-12
265305_at	At2g20340	3.23	Pyridoxal phosphate (PLP)-dependent transferases superfamily protein	1.336E-13
249265_at	At5g41700	3.22	ATUBC8, UBC8, ubiquitin conjugating enzyme 8	1.413E-15
245944_at	At5g19520	3.21	ATMSL9, MSL9, mechanosensitive channel of small conductance-like 9	7.007E-10
265188_at	At1g23800	3.20	ALDH2B, ALDH2B7, aldehyde dehydrogenase 2B7	5.979E-13
262133_at	At1g78000	3.20	SEL1, SULTR1;2, sulfate transporter 1;2	3.125E-11

265216_at	At1g05100	3.20	MAPKKK18, mitogen-activated protein kinase kinase kinase 18	6.727E-08
264289_at	At1g61890	3.19	MATE efflux family protein	1.851E-13
252419_at	At3g47510	3.18	unknown protein; FUNCTIONS IN: molecular_function unknown; INVOLVED IN: biological_process unknown; LOCATED IN: endomembrane system; EXPRESSED IN: 19 plant structures; EXPRESSED DURING: 11 growth stages; Has 15 Blast hits to 15 proteins in 7 species: Archae - 0; Bacteria - 0; Metazoa - 0; Fungi - 0; Plants - 15; Viruses - 0; Other Eukaryotes - 0 (source: NCBI BLink).	1.221E-17
253684_at	At4g29690	3.18	Alkaline-phosphatase-like family protein	9.631E-08
252076_at	At3g51660	3.18	Tautomerase/MIF superfamily protein	1.585E-17
255795_at	At2g33380	3.17	CLO-3, RD20, Caleosin-related family protein	4.371E-16
250781_at	At5g05410	3.17	DREB2, DREB2A, DRE-binding protein 2A	6.535E-14
260676_at	At1g19450	3.15	Major facilitator superfamily protein	1.216E-08
254996_at	At4g10390	3.15	Protein kinase superfamily protein	8.436E-17
263786_at	At2g46370	3.14	FIN219, JAR1, Auxin-responsive GH3 family protein	1.050E-12
259705_at	At1g77450	3.14	anac032, NAC032, NAC domain containing protein 32	7.980E-17
256848_at	At3g27960	3.13	Tetratricopeptide repeat (TPR)-like superfamily protein	1.037E-10
264145_at	At1g79310	3.13	AtMC7, MC7, metacaspase 7	4.761E-05
247717_at	At5g59320	3.11	LTP3, lipid transfer protein 3	8.271E-07
262164_at	At1g78070	3.11	Transducin/WD40 repeat-like superfamily protein	5.182E-11
246235_at	At4g36830	3.11	HOS3-1, GNS1/SUR4 membrane protein family	1.024E-08
249205_at	At5g42600	3.10	MRN1, marneral synthase	1.882E-13
264436_at	At1g10370	3.09	ATGSTU17, ERD9, GST30, GST30B, Glutathione S-transferase family protein	9.101E-09
247109_at	At5g65870	3.09	ATPSK5, PSK5, PSK5, phytosulfokine 5 precursor	1.305E-08
245267_at	At4g14060	3.08	Polyketide cyclase/dehydrase and lipid transport superfamily protein	1.080E-17
253872_at	At4g27410	3.07	ANAC072, RD26, NAC (No Apical Meristem) domain transcriptional regulator superfamily protein	2.721E-20
265058_s_at	At1g52040	3.07	ATMBP, MBP1, myrosinase-binding protein 1	3.468E-21
247026_at	At5g67080	3.07	MAPKKK19, mitogen-activated protein kinase kinase kinase 19	5.392E-16
260205_at	At1g70700	3.06	JAZ9, TIFY7, TIFY domain/Divergent CCT motif family protein	3.876E-15
251480_at	At3g59710	3.06	NAD(P)-binding Rossmann-fold superfamily protein	4.978E-07
248625_at	At5g48880	3.06	KAT5, PKT1, PKT2, peroxisomal 3-keto-acyl-CoA thiolase 2	7.153E-16
246376_at	At1g51950	3.05	IAA18, indole-3-acetic acid inducible 18	2.484E-15
262873_at	At1g64700	3.04	unknown protein; FUNCTIONS IN: molecular_function unknown; INVOLVED IN: N-terminal protein myristoylation; LOCATED IN: cellular_component unknown; EXPRESSED IN: 17 plant structures; EXPRESSED DURING: 11 growth stages; BEST Arabidopsis thaliana protein match is: unknown protein (TAIR:AT3G61920.1); Has 48 Blast hits to 47 proteins in 7 species: Archae - 0; Bacteria - 0; Metazoa - 0; Fungi - 0; Plants - 48; Viruses - 0; Other Eukaryotes - 0 (source: NCBI BLink).	2.992E-04
245275_at	At4g15210	3.04	AT-BETA-AMY, ATBETA-AMY, BAM5, BMY1, RAM1, beta-amylase 5	3.408E-20
248311_at	At5g52570	3.03	B2, BCH2, BETA-OHASE 2, CHY2, beta-carotene hydroxylase 2	1.603E-12
266532_at	At2g16890	3.03	UDP-Glycosyltransferase superfamily protein	6.148E-06
263972_at	At2g42760	3.02	unknown protein; CONTAINS InterPro DOMAIN/s: Protein of unknown function DUF1685 (InterPro:IPR012881); Has 170 Blast hits to 164 proteins in 34 species: Archae - 0; Bacteria - 1; Metazoa - 26; Fungi - 10; Plants - 107; Viruses - 0; Other Eukaryotes - 26 (source: NCBI BLink).	2.829E-18
259653_at	At1g55240	3.01	Family of unknown function (DUF716)	3.973E-13
255484_at	At4g02540	3.01	Cysteine/Histidine-rich C1 domain family protein	6.119E-12
253074_at	At4g36140	3.01	disease resistance protein (TIR-NBS-LRR class), putative	4.445E-07
248395_at	At5g52120	3.00	AtPP2-A14, PP2-A14, phloem protein 2-A14	2.040E-11

[a] *Arabidopsis thaliana* gene identifier code assigned by the Arabidopsis Genome Initiatives (AGI; https://www.arabidopsis.org/portals/nomenclature/).

[b] Mean of two independent biological experiments (*aln-1* versus wild-type).

[c] Gene symbol and description as provided by The Arabidopsis Information Resource (TAIR; release 10; https://www.arabidopsis.org/).

[d] *P*-value determined by a two-way ANOVA.

Table S4. Genes with significantly reduced expression in the *aln-1* mutant.

Listed are those whose changes in transcript levels decreased by equal to or greater than 3-fold with the statistical significance level of 0.001 by a two-way analysis of variance (ANOVA). These genes were selected from the revised microarray data (Gene Expression Omnibus accession number GSE73841) that had been parametrically renormalized using the SuperNORM data service (Skylight Biotech Inc, Akita, Japan), according to the three-parameter lognormal distribution method (Konishi 2004).

Affymetrix probe ID	AGI[a]	Fold change[b]	Gene symbol and description[c]	*P*-value[d]
263174_at	At1g54040	-35.12	ESP, ESR, TASTY, epithiospecifier protein	6.444E-22
253707_at	At4g29200	-34.79	Beta-galactosidase related protein	1.139E-08
266385_at	At2g14610	-32.83	ATPR1, PR 1, PR1, pathogenesis-related gene 1	4.620E-08
266070_at	At2g18660	-28.28	PNP-A, plant natriuretic peptide A	3.414E-07
255437_at	At4g03060	-24.18	AOP2, AOP2 (ALKENYL HYDROXALKYL PRODUCING 2); oxidoreductase, acting on paired donors, with incorporation or reduction of molecular oxygen, 2-oxoglutarate as one donor, and incorporation of one atom each of oxygen into both donors	4.062E-22
261449_at	At1g21120	-18.58	O-methyltransferase family protein	4.834E-06
252345_at	At3g48640	-15.34	unknown protein; FUNCTIONS IN: molecular_function unknown; INVOLVED IN: biological_process unknown; LOCATED IN: endomembrane system; EXPRESSED IN: 10 plant structures; EXPRESSED DURING: 10 growth stages; CONTAINS InterPro DOMAIN/s: Protein of unknown function DUF677 (InterPro:IPR007749); BEST Arabidopsis thaliana protein match is: Protein of unknown function (DUF677) (TAIR:AT5G66670.2); Has 42 Blast hits to 42 proteins in 3 species: Archae - 0; Bacteria - 0; Metazoa - 0; Fungi - 0; Plants - 42; Viruses - 0; Other Eukaryotes - 0 (source: NCBI BLink).	1.603E-04
249082_at	At5g44120	-15.06	ATCRA1, CRA1, CRU1, RmlC-like cupins superfamily protein	6.134E-07
259925_at	At1g75040	-13.69	PR-5, PR5, pathogenesis-related gene 5	3.250E-10
250445_at	At5g10760	-13.55	Eukaryotic aspartyl protease family protein	3.299E-11
253767_at	At4g28520	-13.53	CRC, CRU3, cruciferin 3	5.723E-08
265837_at	At2g14560	-13.32	LURP1, Protein of unknown function (DUF567)	3.411E-10
250476_at	At5g10140	-12.50	AGL25, FLC, FLC, FLF, K-box region and MADS-box transcription factor family protein	3.044E-19
262421_at	At1g50290	-10.78	unknown protein; Has 2 Blast hits to 2 proteins in 1 species: Archae - 0; Bacteria - 0; Metazoa - 0; Fungi - 0; Plants - 2; Viruses - 0; Other Eukaryotes - 0 (source: NCBI BLink).	2.203E-22
246642_s_at	At5g34920	-10.76	transposable element gene	2.470E-06
251344_at	At3g60920	-9.34	CONTAINS InterPro DOMAIN/s: Beige/BEACH (InterPro:IPR000409); BEST Arabidopsis thaliana protein match is: WD-40 repeat family protein / beige-related (TAIR:AT2G45540.1); Has 1795 Blast hits to 1563 proteins in 214 species: Archae - 2; Bacteria - 29; Metazoa - 830; Fungi - 160; Plants - 230; Viruses - 0; Other Eukaryotes - 544 (source: NCBI BLink).	2.455E-08
254574_at	At4g19430	-8.70	unknown protein; FUNCTIONS IN: molecular_function unknown; INVOLVED IN: biological_process unknown; LOCATED IN: chloroplast; EXPRESSED IN: 17 plant structures; EXPRESSED DURING: 13 growth stages; Has 30201 Blast hits to 17322 proteins in 780 species: Archae - 12; Bacteria - 1396; Metazoa - 17338; Fungi - 3422; Plants - 5037; Viruses - 0; Other Eukaryotes - 2996 (source: NCBI BLink).	1.484E-12
251625_at	At3g57260	-8.43	BG2, BGL2, PR-2, PR2, beta-1,3-glucanase 2	2.338E-11
248062_at	At5g55450	-8.29	Bifunctional inhibitor/lipid-transfer protein/seed storage 2S albumin superfamily protein	5.891E-16
263133_at	At1g78450	-8.12	SOUL heme-binding family protein	2.275E-17
257365_x_at	At2g26020	-7.51	PDF1.2b, plant defensin 1.2b	1.537E-06
267546_at	At2g32680	-7.45	AtRLP23, RLP23, receptor like protein 23	5.887E-07
248683_at	At5g48490	-7.27	Bifunctional inhibitor/lipid-transfer protein/seed storage 2S albumin superfamily protein	2.875E-17
252549_at	At3g45860	-7.09	CRK4, cysteine-rich RLK (RECEPTOR-like protein kinase) 4	4.908E-11
265893_at	At2g15040	-7.01	AtRLP18, RLP18, pseudogene, disease resistance protein-related, low similarity to disease resistance protein Cf-2.1 (Lycopersicon pimpinellifolium) GI:1184075; contains Pfam profile PF00560: Leucine Rich Repeat; blastp match of 38% identity and 4.4e-96 P-value to GP|1184075|gb|AAC15779.1||U42444 Cf-2.1 {Lycopersicon pimpinellifolium}	1.419E-12
249777_at	At5g24210	-6.69	alpha/beta-Hydrolases superfamily protein	1.831E-10
247684_at	At5g59670	-6.59	Leucine-rich repeat protein kinase family protein	4.733E-05
264513_at	At1g09420	-5.96	G6PD4, glucose-6-phosphate dehydrogenase 4	9.646E-26
256603_at	At3g28270	-5.94	Protein of unknown function (DUF677)	6.281E-22
258016_at	At3g19350	-5.92	MPC, maternally expressed pab C-terminal	7.141E-09
255653_at	At4g00960	-5.81	Protein kinase superfamily protein	2.461E-05
259385_at	At1g13470	-5.77	Protein of unknown function (DUF1262)	2.084E-05
255879_at	At1g67000	-5.70	Protein kinase superfamily protein	2.648E-06
249890_at	At5g22570	-5.67	ATWRKY38, WRKY38, WRKY DNA-binding protein 38	1.900E-07
263947_at	At2g35820	-5.66	ureidoglycolate hydrolases	2.486E-14
254265_s_at	At4g23140	-5.64	CRK6, cysteine-rich RLK (RECEPTOR-like protein kinase) 6	1.302E-13
267253_at	At2g22960	-5.60	alpha/beta-Hydrolases superfamily protein	6.679E-06
262382_at	At1g72920	-5.52	Toll-Interleukin-Resistance (TIR) domain family protein	9.049E-10
263584_at	At2g17040	-5.43	anac036, NAC036, NAC domain containing protein 36	2.872E-06
258028_at	At3g27473	-5.42	Cysteine/Histidine-rich C1 domain family protein	7.227E-11
248169_at	At5g54610	-5.22	ANK, ankyrin	6.468E-10
256631_at	At3g28320	-4.85	Protein of unknown function (DUF677)	5.048E-07
260568_at	At2g43570	-4.76	CHI, chitinase, putative	1.539E-07
249780_at	At5g24240	-4.67	Phosphatidylinositol 3- and 4-kinase ;Ubiquitin family protein	8.731E-11
249867_at	At5g23020	-4.65	IMS2, MAM-L, MAM3, 2-isopropylmalate synthase 2	6.950E-18
262651_at	At1g14100	-4.60	FUT8, fucosyltransferase 8	1.031E-12
265109_s_at	At1g62630	-4.56	Disease resistance protein (CC-NBS-LRR class) family	1.868E-17
260904_at	At1g02450	-4.52	NIMIN-1, NIMIN1, NIM1-interacting 1	7.944E-07
257139_at	At3g28890	-4.49	AtRLP43, RLP43, receptor like protein 43	1.892E-06

245038_at	At2g26560	-4.45	PLA IIA, PLA2A, PLP2, PLP2, phospholipase A 2A	1.557E-16
267084_at	At2g41180	-4.42	VQ motif-containing protein	9.787E-13
267569_at	At2g30790	-4.38	PSBP-2, photosystem II subunit P-2	1.660E-15
257382_at	At2g40750	-4.36	ATWRKY54, WRKY54, WRKY DNA-binding protein 54	2.989E-15
254253_at	At4g23320	-4.28	CRK24, cysteine-rich RLK (RECEPTOR-like protein kinase) 24	2.008E-07
265063_at	At1g61500	-4.23	S-locus lectin protein kinase family protein	8.497E-14
249645_at	At5g36910	-4.22	THI2.2, thionin 2.2	1.596E-18
245349_at	At4g16690	-4.15	ATMES16, MES16, methyl esterase 16	6.321E-11
265993_at	At2g24160	-4.12	pseudogene, leucine rich repeat protein family, contains leucine rich-repeat domains Pfam:PF00560, INTERPRO:IPR001611; contains some similarity to Cf-4 (Lycopersicon hirsutum) gi|2808683|emb|CAA05268; blastp match of 37% identity and 8.4e-98 P-value to GP|2808683|emb|CAA05268.1||AJ002235 Cf-4 {Lycopersicon hirsutum}	3.359E-10
254805_at	At4g12480	-4.09	pEARLI 1, Bifunctional inhibitor/lipid-transfer protein/seed storage 2S albumin superfamily protein	1.725E-10
246821_at	At5g26920	-4.04	CBP60G, Cam-binding protein 60-like G	3.237E-11
250063_at	At5g17880	-4.00	CSA1, disease resistance protein (TIR-NBS-LRR class)	5.581E-18
266071_at	At2g18680	-3.99	unknown protein; FUNCTIONS IN: molecular_function unknown; INVOLVED IN: biological_process unknown; LOCATED IN: endomembrane system; EXPRESSED IN: male gametophyte, pollen tube; EXPRESSED DURING: L mature pollen stage, M germinated pollen stage; BEST Arabidopsis thaliana protein match is: unknown protein (TAIR:AT2G18690.1); Has 30201 Blast hits to 17322 proteins in 780 species: Archae - 12; Bacteria - 1396; Metazoa - 17338; Fungi - 3422; Plants - 5037; Viruses - 0; Other Eukaryotes - 2996 (source: NCBI BLink).	3.373E-07
266286_at	At2g29170	-3.99	NAD(P)-binding Rossmann-fold superfamily protein	7.009E-12
246603_at	At1g31690	-3.98	Copper amine oxidase family protein	7.832E-21
261221_at	At1g19960	-3.97	BEST Arabidopsis thaliana protein match is: transmembrane receptors (TAIR:AT2G32140.1); Has 41 Blast hits to 41 proteins in 17 species: Archae - 0; Bacteria - 2; Metazoa - 23; Fungi - 0; Plants - 11; Viruses - 0; Other	2.356E-13
245052_at	At2g26440	-3.97	Plant invertase/pectin methylesterase inhibitor superfamily	4.413E-19
250135_at	At5g15360	-3.94	unknown protein; Has 4 Blast hits to 4 proteins in 2 species: Archae - 0; Bacteria - 0; Metazoa - 0; Fungi - 0; Plants - 4; Viruses - 0; Other Eukaryotes - 0 (source: NCBI BLink).	2.920E-10
257625_at	At3g26230	-3.91	CYP71B24, cytochrome P450, family 71, subfamily B, polypeptide 24	5.710E-09
248327_at	At5g52750	-3.88	Heavy metal transport/detoxification superfamily protein	3.025E-16
248848_at	At5g46520	-3.86	Disease resistance protein (TIR-NBS-LRR class) family	1.433E-08
256336_at	At1g72030	-3.86	Acyl-CoA N-acyltransferases (NAT) superfamily protein	1.160E-15
245690_at	At5g04230	-3.81	ATPAL3, PAL3, phenyl alanine ammonia-lyase 3	2.579E-06
253945_at	At4g27050	-3.80	F-box/RNI-like superfamily protein	1.136E-19
257623_at	At3g26210	-3.78	CYP71B23, cytochrome P450, family 71, subfamily B, polypeptide 23	8.243E-17
251705_at	At3g56400	-3.75	ATWRKY70, WRKY70, WRKY DNA-binding protein 70	7.516E-11
253414_at	At4g33050	-3.75	EDA39, calmodulin-binding family protein	1.033E-13
258537_at	At3g04210	-3.67	Disease resistance protein (TIR-NBS class)	1.045E-14
266395_at	At2g43100	-3.65	ATLEUD1, IPMI2, isopropylmalate isomerase 2	9.123E-11
259560_at	At1g21270	-3.63	WAK2, wall-associated kinase 2	1.400E-10
252712_at	At3g43800	-3.52	ATGSTU27, GSTU27, glutathione S-transferase tau 27	3.544E-22
253911_at	At4g27300	-3.49	S-locus lectin protein kinase family protein	2.237E-25
246099_at	At5g20230	-3.46	ATBCB, BCB, BCB, SAG14, blue-copper-binding protein	5.289E-06
252060_at	At3g52430	-3.36	ATPAD4, PAD4, alpha/beta-Hydrolases superfamily protein	7.033E-07
256376_s_at	At1g66690	-3.34	S-adenosyl-L-methionine-dependent methyltransferases superfamily protein	1.219E-06
249843_at	At5g23570	-3.34	ATSGS3, SGS3, XS domain-containing protein / XS zinc finger domain-containing protein-related	2.664E-14
258419_at	At3g16670	-3.33	Pollen Ole e 1 allergen and extensin family protein	4.390E-10
256382_at	At1g66860	-3.32	Class I glutamine amidotransferase-like superfamily protein	3.460E-14
256596_at	At3g28540	-3.28	P-loop containing nucleoside triphosphate hydrolases superfamily protein	4.838E-08
247602_at	At5g60900	-3.28	RLK1, receptor-like protein kinase 1	5.753E-12
260745_at	At1g78370	-3.27	ATGSTU20, GSTU20, glutathione S-transferase TAU 20	1.747E-10
246293_at	At3g56710	-3.27	SIB1, sigma factor binding protein 1	3.155E-11
258277_at	At3g26830	-3.26	CYP71B15, PAD3, Cytochrome P450 superfamily protein	3.761E-05
248975_at	At5g45040	-3.26	Cytochrome c	2.017E-20
249481_at	At5g38900	-3.21	Thioredoxin superfamily protein	2.793E-06
251524_at	At3g58990	-3.21	IPMI1, isopropylmalate isomerase 1	6.946E-13
260396_at	At1g69720	-3.20	HO3, heme oxygenase 3	2.576E-08
254231_at	At4g23810	-3.17	ATWRKY53, WRKY53, WRKY family transcription factor	1.288E-11
258147_at	At3g18070	-3.16	BGLU43, beta glucosidase 43	1.110E-07
245592_at	At4g14540	-3.15	NF-YB3, nuclear factor Y, subunit B3	9.618E-11
263126_at	At1g78460	-3.13	SOUL heme-binding family protein	6.918E-19
248970_at	At5g45380	-3.12	ATDUR3, DUR3, solute:sodium symporters;urea transmembrane transporters	1.316E-16
255184_at	At4g07730	-3.12	transposable element gene	2.513E-06
252977_at	At4g38560	-3.08	Arabidopsis phospholipase-like protein (PEARLI 4) family	1.235E-04
253046_at	At4g37370	-3.07	CYP81D8, cytochrome P450, family 81, subfamily D, polypeptide 8	5.829E-09
264635_at	At1g65500	-3.06	unknown protein; FUNCTIONS IN: molecular_function unknown; INVOLVED IN: biological_process unknown; LOCATED IN: endomembrane system; EXPRESSED IN: 14 plant structures; EXPRESSED DURING: 9 growth stages; BEST Arabidopsis thaliana protein match is: unknown protein (TAIR:AT1G65486.1); Has 23 Blast hits to 23 proteins in 2 species: Archae - 0; Bacteria - 0; Metazoa - 0; Fungi - 0; Plants - 23; Viruses - 0; Other Eukaryotes - 0 (source: NCBI BLink).	8.677E-12
253411_at	At4g32980	-3.06	ATH1, homeobox gene 1	2.344E-12
252411_at	At3g47430	-3.05	PEX11B, peroxin 11B	4.476E-13
258897_at	At3g05730	-3.05	Encodes a defensin-like (DEFL) family protein.	1.976E-07
262381_at	At1g72900	-3.04	Toll-Interleukin-Resistance (TIR) domain-containing protein	8.690E-11
247812_at	At5g58390	-3.04	Peroxidase superfamily protein	2.089E-10
255319_at	At4g04220	-3.01	AtRLP46, RLP46, receptor like protein 46	7.219E-12

245454_at	At4g16920	-3.00	Disease resistance protein (TIR-NBS-LRR class) family	1.522E-08
261782_at	At1g76110	-3.00	HMG (high mobility group) box protein with ARID/BRIGHT DNA-binding domain	1.965E-12

[a] *Arabidopsis thaliana* gene identifier code assigned by the Arabidopsis Genome Initiatives (AGI; https://www.arabidopsis.org/portals/nomenclature/).

[b] Mean of two independent biological experiments (*aln-1* versus wild-type).

[c] Gene symbol and description as provided by The Arabidopsis Information Resource (TAIR; release 10; https://www.arabidopsis.org/).

[d] *P*-value determined by a two-way ANOVA.
